# A telomere-to-telomere gap-free reference genome of *Chionanthus retusus* provides insights into the molecular mechanism underlying petal shape changes

**DOI:** 10.1093/hr/uhae249

**Published:** 2024-09-03

**Authors:** Jinnan Wang, Dong Xu, Ya Lin Sang, Maotong Sun, Cuishuang Liu, Muge Niu, Ying Li, Laishuo Liu, Xiaojiao Han, Jihong Li

**Affiliations:** Shandong Mountain Tai Forest Ecosystem National Station, Key Laboratory of Forest Cultivation in the Lower Yellow River, National Forestry and Grassland Administration, College of Forestry, Shandong Agricultural University, Tai’an 271018, China; Rubber Research Institute, Chinese Academy of Tropical Agricultural Science, Haikou, Hainan 570100, China; Shenzhen Branch, Guangdong Laboratory of Lingnan Modern Agriculture, Genome Analysis Laboratory of the Ministry of Agriculture and Rural Affairs, Agricultural Genomics Institute at Shenzhen, Chinese Academy of Agricultural Sciences, Shenzhen 518120, China; Shandong Mountain Tai Forest Ecosystem National Station, Key Laboratory of Forest Cultivation in the Lower Yellow River, National Forestry and Grassland Administration, College of Forestry, Shandong Agricultural University, Tai’an 271018, China; Shandong Mountain Tai Forest Ecosystem National Station, Key Laboratory of Forest Cultivation in the Lower Yellow River, National Forestry and Grassland Administration, College of Forestry, Shandong Agricultural University, Tai’an 271018, China; Shandong Mountain Tai Forest Ecosystem National Station, Key Laboratory of Forest Cultivation in the Lower Yellow River, National Forestry and Grassland Administration, College of Forestry, Shandong Agricultural University, Tai’an 271018, China; Shandong Mountain Tai Forest Ecosystem National Station, Key Laboratory of Forest Cultivation in the Lower Yellow River, National Forestry and Grassland Administration, College of Forestry, Shandong Agricultural University, Tai’an 271018, China; Shandong Mountain Tai Forest Ecosystem National Station, Key Laboratory of Forest Cultivation in the Lower Yellow River, National Forestry and Grassland Administration, College of Forestry, Shandong Agricultural University, Tai’an 271018, China; Shandong Mountain Tai Forest Ecosystem National Station, Key Laboratory of Forest Cultivation in the Lower Yellow River, National Forestry and Grassland Administration, College of Forestry, Shandong Agricultural University, Tai’an 271018, China; State Key Laboratory of Tree Genetics and Breeding, Key Laboratory of Tree Breeding of Zhejiang Province, Research Institute of Subtropical Forestry, Chinese Academy of Forestry, Hangzhou, Zhejiang 311400, China; Shandong Mountain Tai Forest Ecosystem National Station, Key Laboratory of Forest Cultivation in the Lower Yellow River, National Forestry and Grassland Administration, College of Forestry, Shandong Agricultural University, Tai’an 271018, China

## Abstract

*Chionanthus retusus*, an arbor tree of the Oleaceae family, is an ecologically and economically valuable ornamental plant for its remarkable adaptability in landscaping. During *C. retusus* breeding, we observed diverse floral shapes; however, no available genome for *C. retusus* has hindered the widespread identification of genes related to flower morphology. Thus, a *de novo* telomere-to-telomere (T2T) gap-free genome was generated. The assembly, incorporating high-coverage and long-read sequencing data, successfully yielded two complete haplotypes (687 and 683 Mb). The genome encompasses 42 864 predicted protein-coding genes, with all 46 telomeres and 23 centromeres in one haplotype. Whole-genome duplication analysis revealed that *C. retusus* underwent one fewer event of whole-genome duplication after differentiation compared to other species in the Oleaceae family. Furthermore, flower vein diversity was the main reason for the differences in floral shapes. Auxin-related genes were responsible for petal shape formation on genome-based transcriptome analysis. Specifically, the removal and retention of the first intron in *CrAUX/IAA20* resulted in the production of two transcripts, and the differences in the expression levels of *CrAUX/IAA20* resulted in the variations of flower veins. Compared to transcripts lacking the first intron, transcripts with intron retention caused more severe decreases in the number and length of flower veins in transgenic *Arabidopsis thaliana*. Our findings will deepen our understanding of flower morphology development and provide important theoretical support for the cultivation of Oleaceae.

## Introduction

*Chionanthus retusus* (2n = 2x = 46) is an arbor tree belonging to the family Oleaceae, widely distributed and cultivated in East Asia and North America [1]. In China, it is a famous ornamental plant that is highly adaptable and has a long lifespan, and is an important tree species for afforestation in barren mountains [2]. The leaves and flowers of *C. retusus* are also used as Chinese traditional medicine and tea due to its rich flavonoids, anthocyanins, and tea polyphenols [3, 4, 5]. In the southern region of China, the flowers and leaves of *C. retusus* are commonly utilized to produce a type of tea known as glutinous rice tea [5]. Moreover, *C. retusus*, a native tree species originating from China, is highly suitable for landscaping purposes for its abundant blooming of white flowers in April. Consequently, it is frequently referred to as ‘April Snow’ in China. To date, *C. retusus* has been extensively cultivated as an ornamental tree in gardens, and the variability in its floral morphology has garnered significant attention in the process of selecting and breeding new varieties [6]. Due to its wide array of floral morphologies (Fig. S1), *C. retusus* is well suited for investigating the mechanisms that regulate flower development.

The morphogenesis of lateral organs is closely related to vasculature patterning [7]. Earlier analyses of mutants with various lateral organs with abnormal shapes and vascular systems have provided important insights into the relationship between organ shape and vascular development [8]. Unfortunately, there are only a few published reports describing the association between floral shapes and petal vasculature development. The expression of the *Vigna radiata* gene encoding the plant-specific Dof-like transcription factor LOW (Love On Wings) in *Arabidopsis thaliana* alters the petal vasculature pattern [9]. In the chrysanthemum mutant ‘MADG’, the thickening of the petal vasculature leads to the development of a petal tip that is unable to bend [10]. Therefore, exploring the potential genes regulating petal vasculature variations on flower morphology in *C. retusus* presents an intriguing avenue for research.

Among the *C. retusus* varieties we have bred, ‘Xuezaohua’ (XZH; typical flat petal), ‘Xuedenglong’ (XDL; semi-closed corolla with inward-turning petal), and ‘Xuexuan’ (XX; spiral petal) vary in terms of floral shape. These three varieties are appropriate for clarifying the mechanisms controlling petal shapes and vasculature development due to their phenotypic advantages. However, no available genomic resources for *C. retusus* makes it relatively difficult to elucidate the molecular mechanisms of petal shape formation. Therefore, the assembly of a telomere-to-telomere (T2T)-level genome and the investigation of floral developmental characteristics at the genetic level are essential for understanding the variations in floral traits within different varieties. Additionally, this approach can contribute valuable insights for enhancing the precision of chromosome mapping [11, 12].

In this study, we used PacBio HiFi and Oxford Nanopore Technology (ONT) reads of XZH to assemble the first T2T-level gap-free reference genome of a member of the family Oleaceae and obtain the complete assembly of two haplotypes. Using this reference genome, we conducted a comprehensive morphophysiological analysis of the three selected varieties to identify the key factors regulating important *C. retusus* ornamental traits, including petal vasculature characteristics and cell wall thickness. Furthermore, by annotating genes and examining the transcriptome, we revealed a co-expression network regulating the flower organ developmental stages. Specifically, AUX/IAA20 was identified as an auxin signal transduction-related protein that potentially controls petal shapes by modulating the petal vasculature. Our findings have further clarified the *C. retusus* genome, while also deepening our understanding of the molecular mechanisms underlying petal shape formation.

## Results

### *De novo* assembly and annotation of the gap-free *C. retusus* genome

**Figure 1:**
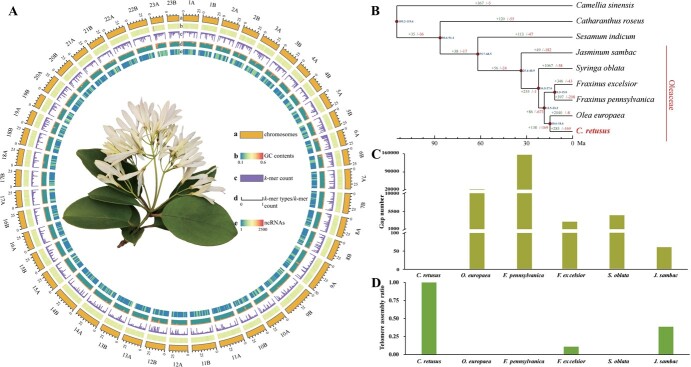
Genome assembly. (A) Two haplotypes of *C. retusus* genome. a, chromosome length in Mb; b, GC contnet; c, *k*-mer count; d, *k*-mer types/*k*-mer count; e, ncRNAs. (B) Evolutionary relationships and divergence times between *C. retusus* and its closely related species. (C) The number of gaps in the published genomes of Oleaceae species. (D) Telomere assembly ratio. Telomere assembly ratio = the number of the assembled telomeres/the total number of telomeres.

 PacBio HiFi reads (41.0 Gb) and chromosome conformation capture sequencing (Hi-C) data (76.0 Gb) were used to assemble the *C. retusus* genome (Table S1). After sequencing, the heterozygosity of the *C. retusus* genome (1.68%) was determined according to the *k*-mer depth distribution (73) (Fig. S2, Table S2). We used the HiC-Pro program to generate chromosomal interaction maps on the basis of Hi-C reads to confirm the correct order and orientation of all pseudomolecules (Table S3, Fig. S3). Considering the high heterozygosity of *C. retusus*, we decided to assemble its two haplotypes separately. Finally, we obtained a gap-free T2T genome with an N50 length of 31.57 Mb. The two haplotypes were 687 and 683 Mb in size, which is close to the estimated genome size of ~757 Mb obtained by *k*-mer survey (Fig. 1A, Table S2). According to the Benchmarking Universal Single-Copy Orthologs (BUSCO) program (using embryophyta_odb10 dataset), the assembly was 99.3% complete, with only 5 of 1614 genes missing (Table S4). Besides, LAI (LTR Assembly Index) value also reached 16.2 (Table S4), indicating that the assembled genome is a high-level reference genome for the studies of *C. retusus*. The 46 chromosomes from the two haplotypes included 14 regions with unknown sequences (gaps), which were filled using HiFi and ultralong ONT (52.7× coverage) data and the Hifiasm (0.19.8-r602) software. Of these 14 gaps, eight gaps were filled directly using the generated contigs. Next, we manually filled the remaining gaps and assembled the two missing telomeres mainly using ONT data. Following the methodology described by Shang et al. [13], we utilized the IGV software to observe the distribution of ONT reads within the post-filled gap regions. The results indicated a favorable continuity in the distribution of ONT reads across the filled gap regions, suggesting the correctness of the gap-filling process (Fig. S4).

After the assembly process was completed, we used NextPolish to further correct the assembly results [14]. A total of 92 telomeres at both ends of 46 chromosomers were completely assembled, and the telomere repeat units were CCCATTT (at the 5′ end) and TTTAGGG (at the 3′ end), which were the same as those of other species. The subsequent comparison between the *C. retusus* genome assembly and the assembled genomes of closely related species (Fig. 1B) revealed that the genomes of the close relatives contained many gaps (61–139 239) (Fig. 1C). Furthermore, the telomere assembly rate was higher for *C. retusus* (100%) than for its close relatives (Fig. 1D). Accordingly, among the examined Oleaceae species, *C. retusus* had the most complete genome assembly.

*De novo*, homology-based, and transcript-based methods were used to annotate the *C. retusus* genome. A total of 42 864 protein-coding genes were predicted (Table S5, S6, and S7). Finally, a total of 41 352 predicted genes (96.47%) were annotated in at least one database (Table S7), with 14 773 genes (34.5%) annotated in all four databases (Fig. S5). The *C. retusus* and *Fraxinus excelsior* genomes had a similar number of annotated genes, whereas the *Olea europaea* genome had fewer annotated genes (Table S6). Additionally, 716 transfer RNAs, 7234 ribosomal RNAs, 2686 small nuclear RNAs, and 731 microRNAs were predicted using Infernal (Table S8). Repetitive sequences accounted for 50.15% of our *C. retusus* genome. More specifically, long-terminal repeat retrotransposons accounted for 39.89% of the genome (Table S9). A total of 18 776 ncRNAs were identified, with chromosomes 9 and 6 carrying the most (1229) and fewest (597), respectively; the average GC content and *k*-mer value were 38.18% and 92.98%, respectively (Table S10).

### Comparative genomic and evolutionary analyses

**Figure 2:**
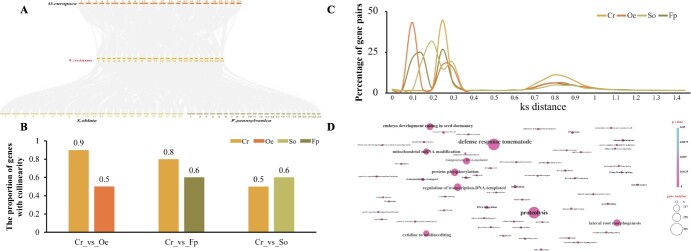
Comparative genomics analysis of *C. retusus* and its closely related species. (A) Collinear analysis of *C. retusus* and its closely related species. (B) The proportion of genes with collinearity in *C. retusus* (Cr), *O. europaea* (Oe), *S. oblata* (So) and *F. pennsylvanica* (Fp). The proportion = the number of collinear genes/total genes. (C) Genome duplication analysis of *C. retusus* and its closely related species. (D) GO enrichment analysis of genes specifically presented in *C. retusus*.

 Chromosome-level genomes of *Jasminum sambac*, *Syringa oblata*, *Fraxinus_excelsior*, *Fraxinus pennsylvanica*, and *O. europaea* in Oleaceae family have been published [15–18]. Because *S. oblata*, *F. pennsylvanica*, and *O. europaea* have the same number of chromosomes as *C. retusus*, we conducted a comparative genomic analysis involving these species. Many conserved genes with syntenic relationships between *C. retusus* and its close relatives were detected (Fig. 2A). The proportion of the genes in each species that were identified as conserved genes was calculated (Fig. 2B). The results reflected the high degree of homology between *C. retusus* and its close relatives. A total of 12 482 *C. retusus* genes lacked syntenic relationships with genes in the close relatives. A Gene Ontology (GO) enrichment analysis of these genes using GFAP [19] indicated they are mainly associated with development (e.g. lateral root morphogenesis and shoot system development), modifications (e.g. protein phosphorylation and positive regulation of histone H3-K4 methylation), and stress responses (e.g. response to wounding and response to freezing) (Fig. 2D).

We constructed a phylogenetic tree using RAxML software and estimated the differentiation time among 13 plant species using PAML software. This analysis utilized 1831 shared single-copy genes identified by OthoMCL software (Fig. 1B). The results revealed that *C. retusus* was most closely related to the Oleaceae species *O. europaea*. The estimated divergence time between *C. retusus* and *O. europaea* is ~14.7 million years ago (MYA) based on different calibration points (Fig. 1B). In the genome of *C. retusus*, 285 and 169 gene families underwent expansion and contraction, respectively (Fig. 1B). In addition, synonymous substitutions per synonymous site (Ks) for the paralogs of *O. europaea*, *Fraxinus pennsylvan*, and *S. oblata*, which experienced a recent whole-genome duplication (WGD) event, and two WGD events occurred in *C. retusus* during evolution (Fig. 2C). The data from Ks, 4dTv value, and collinearity pattern collectively suggested that after differentiating from other species of Oleaceae, *C. retusus* has undergone two rounds of WGD events, which is less than other members (Fig. 2A and C).

### Variations between two haplotypes and centromeric analysis

**Figure 3:**
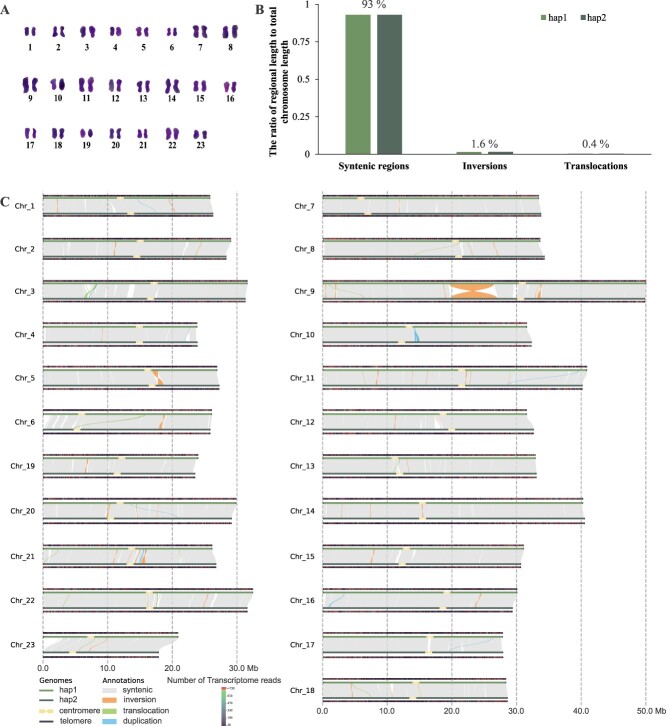
The collinear analysis between the two haplotypes of *C. retusus*. (A) Karyotype map. (B) The length ratio of collinear regions, inversion regions, and translocation regions to the total length of chromosome length. Hap, haplotype. (C) Collinear regions and variations between the two haplotypes. Chr, chromosome.

 Karyotype analysis was conducted on the *C. retusus* genome to provide additional evidence for the accuracy of our assembly results (Fig. 3A). For example, chromosomes 9, 11, and 14 stood out as being longer than the others in the karyotype, which was in accordance with the assembly results (Fig. 3A and 3C). When comparing variations between the two haplotypes, we found that the collinear regions accounted for 93%, while insertions and translocations each accounted for 1.6% and 0.4% of the chromosome length, respectively. Only a few regions, such as chromosome 9, have experienced large-scale inversions (Fig. 3C). The presence of these regions was verified by examining the consistency of ONT reads adjacent to these variant regions and assessing for any aberrant signals (Fig. S6). These results indicated that the two haplotypes still maintain a high degree of homology (Fig. 3B). Therefore, the sequence of haplotype 1 was primarily used for subsequent analysis.

After the assembly of the genome was completed, we used CentIERv1.1 to identify the centromeric regions of *C. retusus*, and 46 centromeres were completely obtained. The accuracy of our predictions is inferred through the systematic examination of high-throughput Hi-C contact frequency data [12]. To further refine the accuracy of our centromere identification, we aligned the transcriptome sequences to the chromosomes to observe regions with expression activity. As shown in Fig. 3C, the centromeric regions corresponded precisely to the chromosomal regions with low expression activity, and this evidence further supports the accuracy of our centromere identification. The analysis of the centromeric regions indicated that most centromeres of *C. retusus* are located in the near-center regions of chromosomes (Fig. 3C). The monomer length of the centromere is 60 bp and is rich in AT content (Table S11). Furthermore, LTRs in centromeric regions are mainly members of the Ty1-copia and Ty3-gypsy superfamilies. Among of them, the numbers of Tork, Ivana, Ale, SIRE, and TAR exceed 100, accounting for 30% of the total LTRs (Table S12).

### Variations in the petal vasculature are responsible for the diversity in *C. retusus* floral shapes

**Figure 4:**
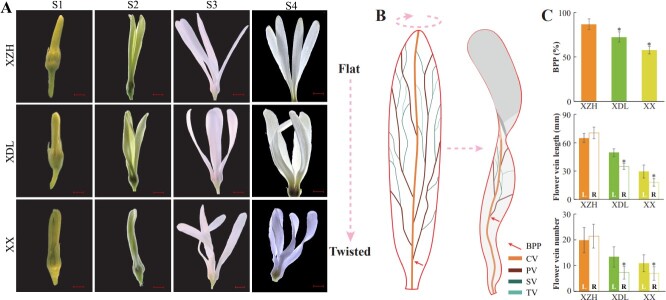
The flower veins of the three *C. retusus* varieties display noticeable distinctions. (A) Flower morphology of *C. retusus* at different development stages. S1: bud stage; S2: initial flowering stage; S3: full flowering stage; S4, final flowering state. Bar = 2 mm. (B) Pattern diagram of petal morphology of *C. retusus*. TV, tertiary vein; PV, primary vein; SV, secondary vein; CV, chief vein; BPP, bifurcation point position. (C) Statistics on the position of bifurcation points and the number and length of flower veins on the left and right sides of petals in three varieties. L, Left; R, Right. Data are presented as means ± SD (*n* = 30). Asterisks indicate significant differences (*t*-test) compared to the XZH and L. ^**^P < 0.01^*^P < 0.05.

 Floral shape is an important trait of *C. retusus*, which is grown as an ornamental garden plant. In this study, we compared three *C. retusus* varieties (XZH, XDL, and XX) that differed regarding the shape of their flowers (Fig. 4A). The flowering process consists of the following four stages: bud stage (S1), initial flowering stage (S2), full flowering stage (S3), and final flowering stage (S4). At S1, there were essentially no differences in floral morphology among the three varieties. At S2, the spiral twisting of petals was detected for all three varieties, but the twisting was more extensive for XX than for the other two varieties. At S3, the flower shapes of all three varieties were well established, with flat petals in XZH, inward-turning petals leading to a semi-closed corolla in XDL, and spiral petals in XX. At S4, the differences in the floral shapes among the three varieties were more pronounced than in the previous stage (Fig. 4A).

To examine the petal vasculature, we conducted tissue transparency experiments at S3. The number of veins and vein thickness were greater for XZH than for XDL and XX (Fig. 4B and Fig. S6A). In contrast, there were no significant differences in the number and length of the main veins among the three varieties (Fig. S7). However, the number and length of the first, second, and third veins were greater for XZH than for XDL and XX (Fig. S7). There were also significant differences in the branching positions of the main and secondary veins as well as in the distribution of the petal vasculature on the left and right sides (Fig. 4B). Specifically, XZH had a larger branching point position (BPP) than XDL and XX (Fig. 4C). Additionally, the number and length of the veins were similar on the left and right sides of the XZH petals (Fig. 4C), whereas the two sides of the XDL and XX petals were asymmetrical, with the number and length of the veins greater on the left side than on the right side of the petals (Fig. 4B and C). These observations suggest that XZH has a strong vasculature, enabling the maintenance of flat petals; the weaker vasculature structures of XDL and XX may be conducive to bending.

### The petal vascular tissue of XZH is significantly stronger than that of the other two varieties

**Figure 5:**
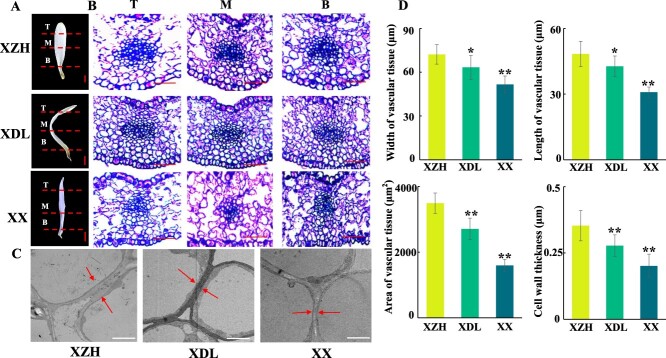
The vascular tissue and cell wall thickness of flower veins of XZH were significantly larger than that of other two varieties. (A) Pattern diagram of paraffin section position. T, Top; M, Middle; B, Base; Bar = 2 mm. (B) Paraffin section showed the vascular tissue morphology of three varieties, Bar = 100 μm. (C) Observation and measure on the cell wall of the main veins of three varieties during the full stage. Bar = 2 μm. (D) Statistics on the length, width, and area of vascular tissue, as well as cell wall thickness in three varieties. Data are presented as means ± SD (*n* = 50). Asterisks indicate significant differences (*t*-test) compared to the XZH. ^*^P < 0.05; ^**^P < 0.01.

 To further clarify the floral vasculature morphological differences among the three varieties, we conducted a histo-anatomical examination of the flower veins (Fig. 5A). The vascular tissue of the main floral vein was significantly thicker for XZH than for XDL and XX. Moreover, the vascular tissue length, width, and area were significantly greater for XZH than for XDL and XX, which was consistent with the observed floral phenotypes (Fig. 5B and D). Furthermore, the XZH vascular tissue had significantly thicker cell walls than the XDL and XX vascular tissues at the S3 stage. Thus, the increased mechanical strength of the XZH flower veins likely contributed to the formation of flat and straight petals (Fig. 5C and D).

### Differentially expressed auxin-related genes responsible for petal shape formation were identified by a comparative transcriptome analysis

 To more precisely characterize the mechanism mediating petal shape formation, with a particular focus on flower vein development, we performed a transcriptome sequencing analysis of the samples collected at four flower development stages. A total of 1621.24 million clean reads were generated from 32 cDNA libraries (Table S13). The Q30 values (sequence error rate of 1%) were >93.17% and the GC content was 42.32%–44.27% (Table S13). According to the PCA, similar samples were clustered together, reflecting the reproducibility of the transcriptome sequencing data (Fig. S8). We subsequently analyzed the differentially expressed genes (DEGs) among XZH, XDL, and XX at different flower development stages (Fig. S9). The XZH_S2 vs XDL_S2 and XZH_S2 vs XX_S2 comparisons respectively detected 9452 and 11 472 DEGs (5569 and 5810 upregulated genes and 3883 and 5662 downregulated genes, respectively). The XZH_S3 versus XDL_S3 and XZH_S3 versus XX_S3 comparisons respectively detected 8767 and 12 621 DEGs (5514 and 8063 upregulated genes and 3253 and 4558 downregulated genes, respectively) (Fig. S9).

**Figure 6:**
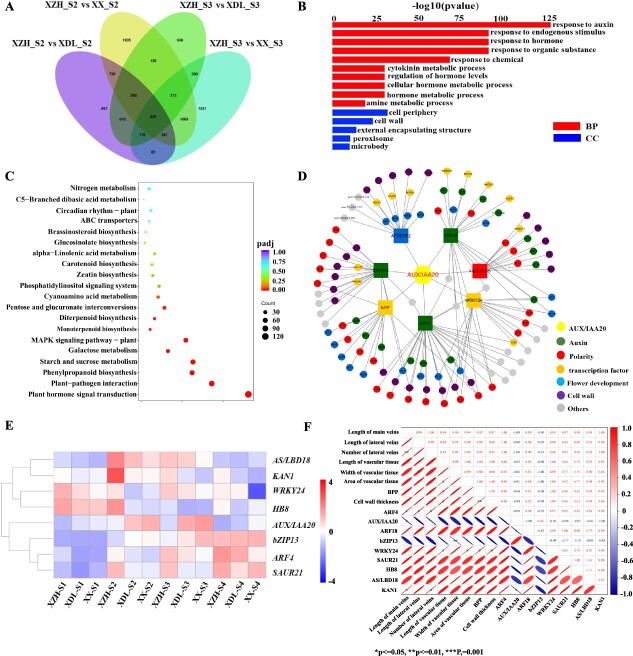
The formation of different flowering morphology. (A) Venn diagram indicates the number of DEGs in four comparisons. (B) GO enrichment analysis of DEGs in four comparisons. (C) KEGG enrichment analysis of DEGs in four comparisons. (D) Subnetwork for evm.TU.Chr17.1287_*CrAUX/IAA20*, low values to small size circle. Different colors represent different types of genes. (E) Expression patterns of hub genes related to flower vein development. (F) Correlation analysis between candidate genes and petal shape phenotype. The asterisks (*) indicate significant differences at *P* < 0.05.

To identify the main genes associated with petal morphology, petal morphology-related DEGs detected by the four comparisons were analyzed. We identified 825 DEGs that were common to all four comparisons (XZH_S2 vs XDL_S2, XZH_S2 vs XX_S2, XZH_S3 vs XDL_S3, and XZH_S3 vs XX_S3) (Fig. 6A). The enriched GO terms among these common genes included the following: response to auxin, response to endogenous stimulus, response to hormone, and response to organic substance (Fig. 6B). There were several enriched Kyoto Encyclopedia of Genes and Genomes (KEGG) pathways among these genes, including plant hormone signal transduction, phenylpropanoid biosynthesis, and MAPK signaling pathway–plant (Fig. 6C).

On the basis of the enriched GO terms and KEGG pathways related to hormones, we further identified 10 gene families related to auxin involved in flower vein development, including auxin-related gene families (*YUCCA*, *AUX/IAA*, *ARF*, *SAUR*, *GH3*, and *PIN*) and polarity-related gene families (*HB*, *KAN*, *YABBY*, and *AS/LBD*) (Fig. S10). With the exception of the *AS/LBD* gene family, the polarity-related genes were mainly expressed at the S1 stage and the S2 stage, after which their expression levels gradually decreased (Fig. S10 and Table S14). The auxin-related genes were primarily expressed during the S2 and S3 stages. In addition, with the exception of the *AUX/IAA* gene family members, the auxin-related genes were highly expressed in XZH, further suggesting auxin promotes flower vein development in *C. retusus*. The *AUX/IAA* genes, which encode auxin signaling pathway inhibitors, were expressed at significantly higher levels in XDL and XX than in XZH (Fig. S10 and Table S14). These expression patterns may indicate that auxin-related genes and their target genes may be important for flower morphogenesis in *C. retusus*.

According to their expression levels, we identified eight auxin-related candidate genes for further analyses (Fig. 6D). Of these genes, *HB8*, *KAN1*, *AS/LBD18*, and *WRKY24* were highly expressed in the early stage of flowering before gradually decreasing as the flowering process progressed. Notably, with the exception of *AUX/IAA20* and *bZIP13*, these genes were expressed at much higher levels in XZH than in XDL and XX (Fig. 6E and Table S15). We also constructed a regulatory network on the basis of the eight auxin-related candidate genes using Cytoscape (Fig. 6D). According to this regulatory network, auxin response factors (*ARFs*) and *AUX/IAA20* control flower vein formation by regulating the expression of genes related to auxin, polarity, and vascular tissues, thereby affecting the *C. retusus* petal shape. The correlation between the expression patterns of the auxin-related candidate genes and flower vein traits indicated that *AUX/IAA20* and *ARFs* are significantly negatively and positively correlated with flower vein traits, respectively (Fig. 6F). Twelve candidate genes were selected and their expression patterns were validated (Fig. S11).

### *CrAUX/IAA20* is a key gene controlling the *C. retusus* petal shape

**Figure 7:**
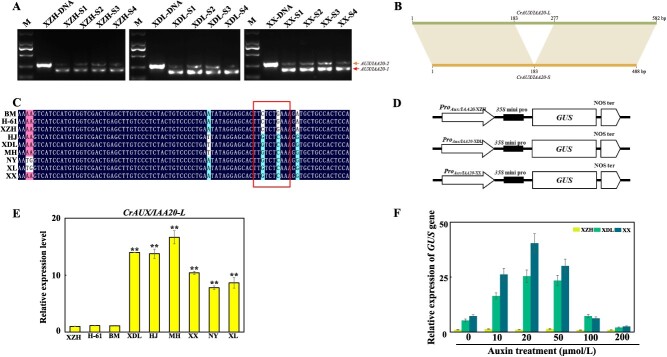
*CrAUX/IAA20* as a key gene in the petal shape of *C. retusus*. (A) Two transcripts of *CrAUX/IAA20* in *C. retusus* flowers. S1: bud stage; S2: initial flowering stage; S3: full flowering stage; S4: final flowering stage; M: marker 2000; long transcript, *CrAUX/IAA20-L*; short transcript, *CrAUX/IAA20-S*. (B) Two transcript sequences (*CrAUX/IAA20-L* and *CrAUX/IAA20-S*) of the *CrAUX/IAA20* gene have been identified, one of which retains an intron sequence. (C) Mutation of AuxRE element in the *CrAUX/IAA20* gene promoter of varieties with flat petals. (D) Construction of promoter vectors. (E) Expression patterns of *CrAUX/IAA20-L* in the petals of *C. retusus* with different flower forms during peak flowering state. The expression level of *CrAUX/IAA20-L* in the XZH was set as 1. *UBC2* was used as an internal control. Data are presented as means ± SD (*n* = 3). Asterisks indicate significant differences (*t*-test) compared to the XZH. *P < 0.05; **P < 0.01. (F) The mutation in the AuxRE element of the XZH gene promoter renders the *AUX/IAA20* promoter insensitive to auxin. The expression of *GUS* in the XZH with 0 μmol/l IAA (0) was set as 1. Data are presented as means ± SD (*n* = 3). Asterisks indicate significant differences (*t*-test) compared to the XZH at each concentration. ^*^P < 0.05; ^**^P < 0.01.

 The examination of the three varieties detected two transcripts of evm.TU.Chr17.1287, which is a homolog (*CrAUX/IAA20*) of the *Arabidopsis AUX/IAA20* gene (Fig. 7A and B). However, only one *AtAUX/IAA20* transcript is produced in *A. thaliana*. Thus, the two transcripts were cloned and sequenced (Fig. 7B). The removal and retention of the first intron in *CrAUX/IAA20* resulted in the production of two transcripts (Fig. 7B and Fig. S12), namely *CrAUX/IAA20-L* (i.e. long transcript encoding 193 amino acids) and *CrAUX/IAA20-S* (i.e. short transcript encoding 161 amino acids) (Fig. 7A and 7B). Interestingly, only *CrAUX/IAA20-S* was detected in the young leaves and stems (Fig. S13). These findings indicated that *CrAUX/IAA20-L* was expressed exclusively in the flowers, suggesting its importance for flowering. Moreover, the *CrAUX/IAA20-L* expression level was significantly higher in XDL and XX than in XZH; the lowest *CrAUX/IAA20-L* expression level was detected in XZH at S3 (Fig. 7E).

The comparison of the *CrAUX/IAA20* promoter sequences in the three varieties revealed mutations in the AuxRE element of *ProCrAUX/IAA20-_XZH_* (Fig. 7C). The AuxRE element is an auxin response element that serves as a binding site for ARFs [20]. Therefore, the mutation in the AuxRE element of *ProCrAUX/IAA20-_XZH_* may modulate the regulatory effect of auxin on *CrAUX/IAA20* expression. Furthermore, we inserted the *ProCrAUX/IAA20* sequences from the three varieties into GUS vectors (Fig. 7D). The *GUS* expression level was highest when *ProCrAUX/IAA20-_XX_* was used as the promoter. The *GUS* expression level was significantly lower when *ProCrAUX/IAA20-_XZH_* served as the promoter. Treatments with different auxin concentrations rapidly increased *GUS* transcription under the control of *ProCrAUX/IAA20-_XDL_* and *ProCrAUX/IAA20-_XX_* whereas they did not substantially alter *GUS* transcription under the control of *ProCrAUX/IAA20-_XZH_* (Fig. 7F). These results indicate that *ProCrAUX/IAA20-_XZH_* was significantly less active than *ProCrAUX/IAA20-_XDL_* and *ProCrAUX/IAA20-_XX_* Furthermore, the insensitivity of *ProCrAUX/IAA20-_XZH_* to auxin was likely due to its mutated AuxRE element.

### Overexpression of *CrAUX/IAA20-L* and *-S* can regulate the petal vasculature of transgenic *A. thaliana*

**Figure 8:**
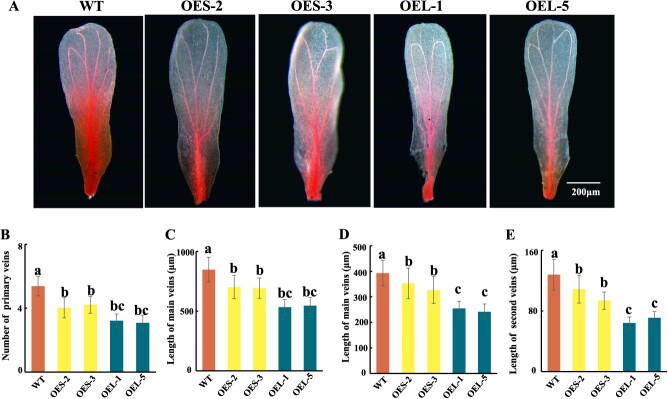
Overexpression of *CrAUX/IAA20-S* and *CrAUX/IAA20-L* can affect the floral veins of *Arabidopsis*. (A) Overexpression of *CrAXU/IAA20-S* and *CrAUX/IAA20-L* can affect the development of floral veins in *Arabidopsis,* Bar = 200 μm; (B), (C), (D), and (E) Number and length of floral veins in WT, *CrAXU/IAA20-S* and *CrAUX/IAA20-L*. Data are presented as means ± SD (*n* = 30). Lowercase letters indicate significant differences between different products. *P* < 0.05.

 There were no significant differences in the small flowers of the transgenic and wild-type (WT) *A. thaliana* plants (Fig. S14), but the flower veins were significantly weaker in the transgenic plants than in the WT plants. More specifically, the flower veins were weakest in the *CrAUX/IAA20-L*-overexpressing plants, followed by the *CrAUX/IAA20-S*-overexpressing plants (Fig. 8A). Additionally, compared with the WT plants, the transgenic plants had fewer and shorter flower veins (Fig. 8B, C, D, E). The expression of auxin-related genes (e.g. *AtARF*, *AtHB*, and *AtSAUR*) was significantly inhibited in the transgenic *A. thaliana* petals (Fig. S15), indicating *CrAUX/IAA20* has important effects on *C. retusus* petal veins.

## Discussion

 In the present study, we generated the first T2T-level gap-free *C. retusus* genome assembly. This high-quality *C. retusus* reference genome will be valuable resource for developing novel *C. retusus* cultivars and for research on the molecular mechanisms underlying flower development as well as evolution.

The diversity in flower shapes influences the ornamental value of plants. Hence, clarifying the mechanism controlling floral shapes is critical for optimizing the breeding of new varieties of ornamental plants. Earlier investigations functionally characterized the genes encoding relevant enzymes and transcription factors affecting petal morphogenesis [21–23]. However, these studies mainly focused on the effects of petal cell proliferation and expansion on petal morphology, with relatively few studies exploring the petal vasculature. We conducted morphological and cytological analyses of the petals of three *C. retusus* varieties and determined that differences in flower shapes were mainly associated with changes in the petal vasculature (Fig. 4, Fig. 5, and Fig. S7). Vasculature patterning reportedly regulates the morphogenesis of lateral organs [7]. Thus, the petal vasculature pattern may affect petal morphogenesis. The thickening of the petal vasculature in the chrysanthemum mutant ‘*MADG*’ prevents the petal tip from bending [10]. Here, we detected significant differences in the petal vasculature among the three *C. retusus* varieties with different flower shapes. Compared with the flowers of the other two varieties, the XZH flowers (flat petals) had more and longer veins (Fig. 4 and Fig. S7). These findings were supported by the examination of paraffin sections and ultrathin sections (Fig. 5B and C). Hence, the relatively strong petal vasculature of the XZH flowers may provide the mechanical support required for the flat petal phenotype. Therefore, the strength of the petal vasculature system has crucial effects on petal morphology.

Lateral organ morphogenesis depends on the development of the vasculature [7]. There were clear differences among the XZH, XDL, and XX inflorescences in terms of their petal vasculature, with more lignified vessels and distinct vein patterning in XZH (Fig. 4, Fig. 5, and Fig. S6). The DEGs detected by the transcriptome analysis of the morphologically diverse flowers were mainly involved in hormone-related pathways (e.g. response to auxin and plant hormone signal transduction) (Fig. S10 and Fig. 6). Earlier studies indicated auxin plays an important role in the development of plant veins [24, 25]. The auxin canalization model, which can be used to explain leaf vein development [26, 27], includes a feedback loop composed of several genes (e.g. *ARF*, *AtHB8*, *AUX/IAA*, and *PIN*) [28]. Auxin activates *ARF* expression, which leads to the activated expression of the intermediate-loop *AUX/IAA* genes. Both ARFs and AUX/IAAs regulate *AtHB8* expression, ultimately linking the auxin signal to vasculature formation [29–31]. In our study, *ARF* homologs, which are believed to encode proteins that induce the formation of vascular strands, were more abundantly transcribed at S2 and S3 in XZH than in the other two varieties (Fig. S10). Hence, we hypothesized that increases in *ARF* transcription promote vascular development. This hypothesis was supported by the fact many genes encoding proteins that were predicted to function downstream of the ARFs (e.g. *AtHB8*, *SAUR*, *GH3*, and *PIN*) were differentially transcribed at S2 and S3 among XZH, XDL, and XX, consistent with the changes in the expression of *ARF* homologs (Fig. S10).

The *AUX/IAA* genes encode inhibitors in the feedback loop. Previous studies showed AUX/IAA proteins suppress *ARF* activities and are degraded when auxin concentrations reach peak levels [32–36]. Similarly, *AUX/IAA* expression was significantly enriched in XDL and XX at S2 and S3, which was in contrast to the changes in *ARF* abundance (Fig. S10). Accordingly, the ARF-AUX/IAA-HB module is likely involved in the induction of petal morphogenesis. We subsequently identified *CrAUX/IAA20* as a member of the *AUX/IAA* gene family that encodes a regulator of the *C. retusus* petal shape. Notably, *CrAUX/IAA20-L* was detected only in the flowers (Fig. 7A and B, Fig. S13). Furthermore, *CrAUX/IAA20-L* was expressed at significantly lower levels in XZH than in XDL and XX, possibly because the mutation in the AuxRE element of *ProCrAUX/IAA20*-_XZH_ resulted in auxin insensitivity (Fig. 7C and D). According to earlier research, *AUX/IAA* is essential for leaf vein development [37]. In the *A. thaliana axr6* mutant, the lack of a functional AXR6 prevents the normal assembly of the SCF, thereby inhibiting the degradation of AUX/IAA7, resulting in a decrease in the number of leaf veins [38, 39]. Other studies demonstrated that AUX/IAA12 can modulate leaf vein development by inhibiting the expression of *ARF* genes [40–42]. In the *zmiaa28* maize mutant, because of the mutation to *ZmIAA28*, there are no intermediate veins in the leaves [43]. Here, the overexpression of *CrAUX/IAA20* in *A. thaliana* petals inhibited the expression of multiple genes (e.g. *AtARF* and *AtHB*), thereby hindering petal vascular tissue development (Fig. 8A, Fig. S14, and Fig. S15). This result is similar to the effects of *AUX/IAAs* on leaf veins, suggesting that the regulatory effects of the ARF-AUX/IAA-HB module on vascular tissue development are conserved. In this study, we used a newly assembled genome along with the transcriptome data for different flowering stages to develop a co-expression network that controls petal morphogenesis and to predict the contributions of key transcription factors. Our results suggest that *ARFs* and *AUX/IAAs* coordinately regulate vascular tissue development, which is in accordance with the findings in other species [27, 40, 42]. These hub genes play a vital role in ensuring *C. retusus* flower formation normally. Clarifying their interactions may provide valuable insights into plant development.

In conclusion, the T2T gap-free genome assembly that was generated and validated in this study represents a long-awaited key resource for the ornamental plant species *C. retusus*. This genomic resource may be useful for characterizing genetic mutations and for identifying new genes. In addition, key genes controlling petal shape changes were identified, including *CrAUX/IAA20*. The T2T gap-free reference genome should be combined with the transcriptome data for future genetic analyses as well as for the genetic improvement of *C. retusus*.

## Materials and methods

### Plant materials

 A 5-year-old *C. retusus* XZH plant with stable flower traits was obtained from the nursery of Shandong Agriculture University (Shandong province, China) for the genome sequencing analysis and the *de novo* assembly of the genome. Fresh leaves were collected and used to prepare the genome sequencing library. The RNA-seq data for the XZH stems, leaves, roots, buds, and xylem were used to annotate the genome. Flowers at four development stages (Fig. 4A) were collected from three varieties (XZH, XDL, and XX), with three replicates per stage, for the transcriptome sequencing analysis. We determine the four stages of flower development according to the following criteria. The bud stage (S1): 30% of the flower buds are exposed, and the rest are still closed; the initial flowering stage (S2): the flowers bloom slightly, but most are still in a semi-closed state; the full flowering stage (S3): >50% of the flowers are open; final flowering stage (S4): 70% or almost all of them are open. Plant materials were immediately frozen in liquid nitrogen and stored at −80°C prior to extracting genomic DNA and RNA.

### Genome assembly

 We performed the *de novo* assembly by referring to the method described by Shang et al [13]. DNA was extracted using a plant genomic DNA kit from TIANGEN (DP305, Beijing, China), and its corresponding library was generated using a NEBNext Ultra II DNA Library Prep Kit for Illumina (Massachusetts, USA). For PacBio HiFi library construction, >5 μg of sheared DNA was subjected to size selection on a BluePippin instrument (Massachusetts, USA). The library was loaded in SMRT Cells using DNA Sequencing Reagent Kit, and the SMRT cells were run on a PacBio RSII-CCS system. The Ultralong ONT sequencing library was prepared according to the Nanopore protocol. The Hi-C library was constructed from young leaves by the Novogene Corporation Inc. (Beijing, China) using a previously described technique [44]. On the basis of the HiFi, Hi-C, and ultralong ONT data, the *C. retusus* genome was assembled using the default parameters of Hifiasm. The ordering and orientation of Hi-C data were modified using Juicebox to ensure their accuracy.

### Gap filling and assembly integrity assessment

 After assembling the chromosome-level genome, we used HiFi and ONT reads to fill gaps manually. Specifically, HiFi reads were aligned to the assembled genome using Minimap2 to pinpoint reads aligning in proximity to gaps. Subsequently, the assembly relationship between reads was established by aligning ONT reads with HiFi reads, enabling the construction of an assembly path to systematically fill the gaps. To aid in the selection of alignment results, we calculated the number of unique *k*-mers (a *k*-mer that appears only once throughout the entire genome) by utilizing Jellyfish. Additionally, we employed RAviz to enhance the efficiency of selecting alignments [45]. To fill any gaps and assemble the missing telomeres, the default parameters of Minimap2 were used to align sequences, whereas Jellyfish was used for analyzing *k*-mers. RAviz was used to visualize the gaps and sequence alignments [45, 46].

After obtaining the T2T level genome, we employed BUSCO and CentIERv1.1 pipeline [47], respectively, to calculate the BUSCO score and LAI value of the genome, in order to assess the completeness of the genome assembly [48, 49].

### Collinearity analysis

 The collinearity between haplotypes 1 and 2 was analyzed using SyRI [50], after which we mapped the transcriptome reads for the flower organs to the *C. retusus* chromosomes using the default parameters of Minimap2 [46]. The alignment results were filtered as follows: mapping quality ≥60 and alignment length/query sequence length ≥80%. The number of retained reads was determined according to alignment positions using a sliding window approach (10 kb step size).

### Centromere analysis

 We used CentIER to examine the *C. retusus* centromeres; specific details regarding this tool are available online (https://github.com/simon19891216/CentIER) [47]. After the gaps were filled, the genome sequence was analyzed using the default parameters of CentIER. Based on the CentIER results, we have conclusively identified the centromeric region in conjunction with Hi-C signals (https://github.com/ruoyu1123/Gut-HiC/blob/main/scripts/hicplotter.py).

### Gene functional annotation

 Genes were annotated as previously described [51]. Using the encoded protein sequences in *C. retusus* and the GFAP program [19], the genes were functionally annotated according to the GO, KEGG, Pfam, and Nr databases, with an *e*-value threshold of 1e^−5^ (i.e. default value).

### Karyotype analysis

 Karyotype analysis was conducted as previously described [52]. The root tips of XZH were extensively washed with distilled water and subsequently submerged in a pretreatment solution consisting of a saturated p-dichlorobenzene and 2 mmol/l 8-hydroxyquinoline (in a ratio of 1:1 v/v). After the 6-h pretreatment, the materials were thoroughly rinsed 3–5 times with distilled water. Subsequently, they were fixed in Carnot’s solution of acetic acid- anhydrous ethanol (1:3, v/v) and stored at 4°C. After 24 h, the materials were immersed in 1 mol/l HCl, and incubated at 60°C for 15 min for dissociation. Then, they were stained using a magenta dye solution and observed under a 100× microscope (Nikon 80i, Japan). The images were processed using Photoshop 2020 to segment chromosomes and facilitated homologous chromosome pairing. Furthermore, data organization and karyotype pattern construction were performed using Microsoft Excel 2019.

### Examination of the secondary vascular tissue and cell wall thickness of petals

 For the flower vein staining experiment, the petals of three varieties (XZH, XDL, and XX) that were collected during the S3 stage were fixed in FAA solution and then transferred to a saturated trichloroacetaldehyde solution until they were completely transparent. The petals were stained with a 1% safranine solution for 30 min and then the flower vein structures were examined using the 80i microscope (Nikon, Japan). To more precisely characterize the petal vasculature, we determined the BPP by dividing the distance from the first bifurcation point in the lower part of the main vein to the top of the petals by the total petal length. The flower vein length, number, and BPP were determined using the ImageJ software. The flower veins of at least 30 petals from three independent plants were measured and then analyzed using Student’s *t*-test.

The upper, middle, and lower parts of the petals at the S3 stage were fixed in FAA solution and then dehydrated in a series of ethanol solutions with increasing concentrations. Finally, the petals were embedded in paraffin and transversely sectioned (10 μm thickness) using the EM UC7 microtome (Leica, Germany). Sections were stained with 0.01% (w/v) Toluidine Blue O, after which digital images were captured using the 80i microscope. To examine the petal vasculature microstructure, the middle parts of the petals collected at the S3 stage were manually cut into ~2-mm sections, which were then fixed in a solution comprising 4.0% (v/v) glutaraldehyde and 0.1 M phosphate buffer (pH 6.8) for >24 h. The sections were subsequently treated with osmic acid, dehydrated, embedded in Epon resin, and polymerized. The samples were cut into 70-nm-thick sections using the EM UC6 ultramicrotome (Leica) and then stained with uranium acetate and lead citrate. The sections were examined at an accelerating voltage of 120 kV using the Talos L120C G2 transmission electron microscope (Thermo Fisher, USA). The vascular tissue length, width, and area and the cell wall thickness were measured using microscopy images and the ImageJ software. More specifically, the vascular tissue length, width, and area and the cell wall thickness of at least 50 cells from three independent plants were measured and then analyzed using Student’s *t*-test.

### Transcriptome sequencing, gene expression analysis, and co-expression regulatory network analysis

 The transcriptome sequencing analysis was performed by Beijing Novogene Co. Ltd. Samples were collected at four flower development stages (S1 to S4) for the three varieties. Total RNA was extracted using a plant RNA kit from TIANGEN (DP432, Beijing, China), and using the RNA Nano 6000 Assay Kit for the Bioanalyzer 2100 system, and RNA-Seq libraries were constructed according to the protocol provided by Illumina and sequenced on the NovaSeq6000 platform. The RNA-seq short reads obtained for 36 tissue samples were aligned to our chromosome-scale genome using HISAT2 [53]. The read counts for these samples were calculated using HTSeq. Differential gene expression was analyzed using the R package DESeq2. The following criteria were applied to detect significant DEGs: |log_2_(fold-change)| > 1 and adjusted *P* < 0.05. To assess the relationships between DEGs, we constructed a co-expression regulatory network using the following parameters of the WGCNA package [54]: weighted network, unsigned, hierarchical clustering tree, dynamic hybrid tree cut algorithm, power 8, and minModuleSize = 30. The network was visualized using Cytoscape (v3.5.1). Network statistics were calculated using the NetworkAnalyzer tool in Cytoscape [55]. Gene expression heat maps were generated using the default parameters of an online program (https://magic.novogene.com/customer/main#/small-tools/2ec0f5f4ac89941fe4fb43 480001f375/1/heatmap).

### Vector construction and plant transformation

 The *CrAUX/IAA20* promoter region (1500 bp upstream of the ATG start codon) was amplified from the genomic DNA of the three *C. retusus* varieties using gene-specific primers and then cloned into the pBI121 expression vector. *Agrobacterium tumefaciens* (GV3101) cells harboring the recombinant expression vectors were used for the transient transformation of tobacco leaves. At 3 days post-infection, the tobacco leaves were treated with deionized water and various indole-3-acetic acid (IAA) concentrations. Following the extraction of RNA, the *GUS* expression level was determined by performing a quantitative real-time PCR (qRT-PCR) analysis. The primers used for cloning the promoter region and for the qRT-PCR analysis are listed in Table S16.

The full-length coding sequences of *CrAUX/IAA20-L* and *CrAUX/IAA20-S* were amplified from the floral cDNA of XZH, XDL, and XX using gene-specific primers and then cloned into pRI101 vectors. The recombinant vectors were sequenced to confirm the incorporated sequences were correct. They were then used for the genetic transformation of *A. thaliana* Col-0 plants according to the floral-dip method [56]. After extracting RNA, the qRT-PCR analysis was performed using SYBR Premix Ex Taq™ II (Takara, Dalian, China) and the CFX96 Real-Time PCR System (BioRad, USA). Three biological replicates and three technical replicates were used to analyze each gene. Relative expression levels were calculated according to the comparative 2^−ΔΔCt^ method. The primers used for cloning genes and for the qRT-PCR analysis are listed in Table S16.

## Supplementary Material

Web_Material_uhae249

## Data Availability

The Hi-C and ONT data, as well was the assemblies have been deposited to National Genomics Data Center with Bioproject ID of CRA011999. The RNA-seq data have been deposited to China National GeneBank DataBase with Bioprojrct ID of CNP0005081.
