# Supplementary material for: A telomere-to-telomere gap-free reference genome of Chionanthus retusus provides insights into the molecular mechanism underlying petal shape changes
Source: Hortic Res. 2024 Sep 3;11(12):uhae249. doi: 10.1093/hr/uhae249 (PMC11629972; doi:10.1093/hr/uhae249)
Supplement: Web_Material_uhae249 [file web_material_uhae249.zip › Supplementray.docx]

A telomere-to-telomere gap-free reference genome of *Chionanthus retusus* provides insights into the molecular mechanism underlying petal shape changes

Jinnan Wang^1#^, Dong Xu^2,4#^, Yalin Sang^1#^, Maotong Sun^1^, Cuishuang Liu^1^, Muge Niu^1^, Ying Li^1^, Laishuo Liu^1^, Xiaojiao Han^3*^, Jihong Li^1*^

*^1^Shandong Mountain Tai Forest Ecosystem National Station, Key Laboratory of Forest Cultivation in the Lower Yellow River, National Forestry and Grassland Administration, College of Forestry, Shandong Agricultural University, Tai’an, 271018, China*

*^2^Rubber Research Institute, Chinese Academy of Tropical Agricultural Science, Haikou, Hainan, 570100, China*

*^3^State Key Laboratory of Tree Genetics and Breeding, Key Laboratory of Tree Breeding of Zhejiang Province, Research Institute of Subtropical Forestry, Chinese Academy of Forestry, Hangzhou, Zhejiang, 311400, China*

*^4^Shenzhen Branch, Guangdong Laboratory of Lingnan Modern Agriculture, Genome Analysis Laboratory of the Ministry of Agriculture and Rural Affairs, Agricultural Genomics Institute at Shenzhen, Chinese Academy of Agricultural Sciences, Shenzhen, 518120, China*

#These authors contributed equally to this work.

*Correspondence: Xiaojiao Han ([hanxiaojiao1004@163.com](mailto:hanxiaojiao1004@163.com)), Jihong Li (jhli@sdau.edu.cn)

**Supplemental Figures**


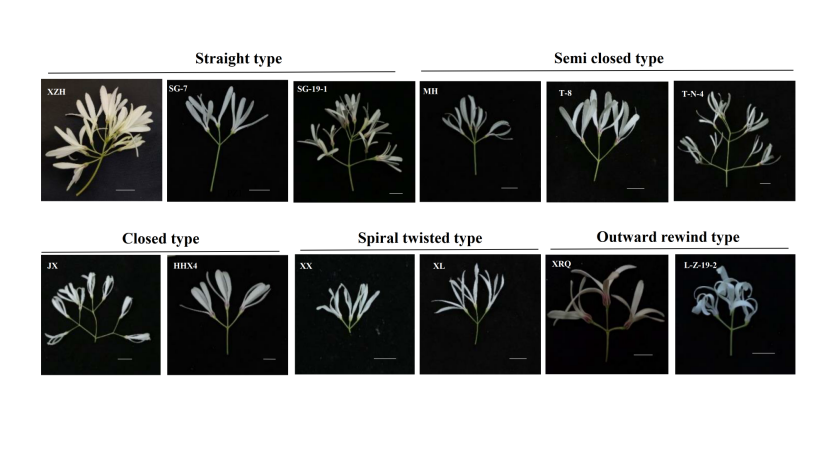


**Supplemental** Figure S1 The flower morphology of *Chionanthus retusus* exhibits rich variations.

XZH, *C. retusus*‘Xuezaohua’; SG-7, *C. retusus* ‘Xueqingxiang’; SG-19-1, *C. retusus* ‘SG-19-1’; MH, *C. retusus* ‘Muhuang’; T-8, *C. retusus* ‘T-8’; T-N-4, *C. retusus* ‘T-N-4’; JX, *C. retusus* ‘Jingxue’; HHX4, *C.retusus* ‘H-Huaxiang4’; XX, *C. retusus* ‘Xuexuan’; XL, *C. retusus* ‘Xueluo’; XRQ, *C. retusus* ‘Xuerongqiu’; L-Z-19-2, *C. retusus*‘L-Z-19-2’.


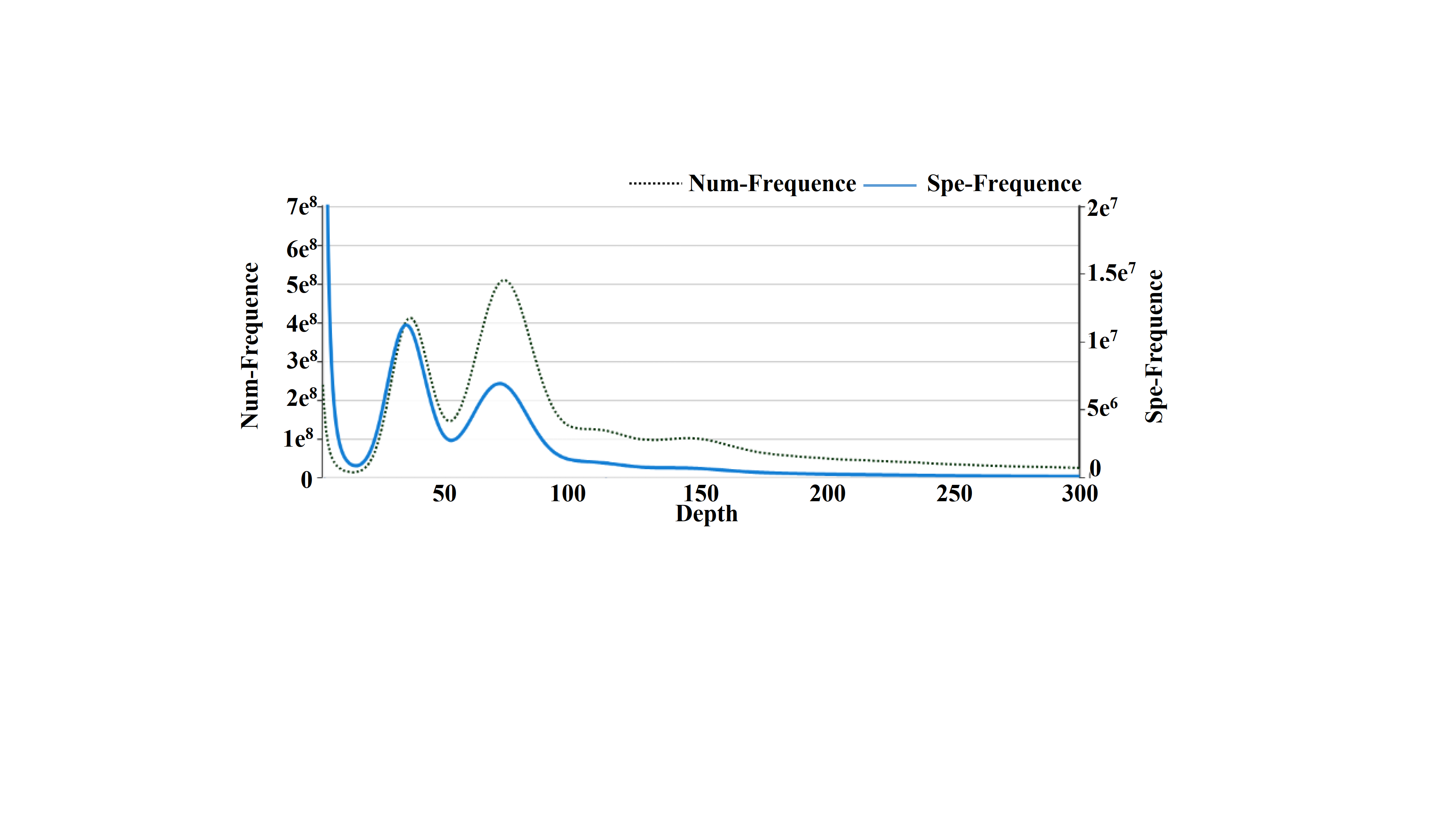


**Supplemental** Figure S2 *k*-mer analysis for estimating the genome size of *C. retusus*


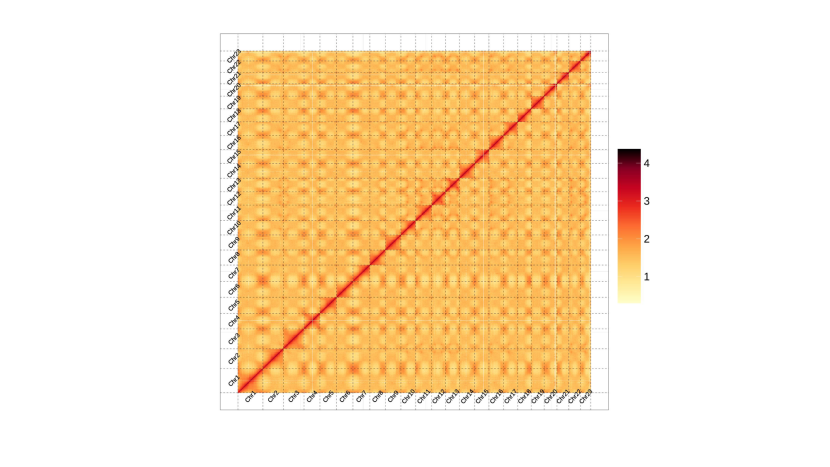


**Supplemental** Figure S3 Hi-C interaction heatmap of the *C. retusus* genome


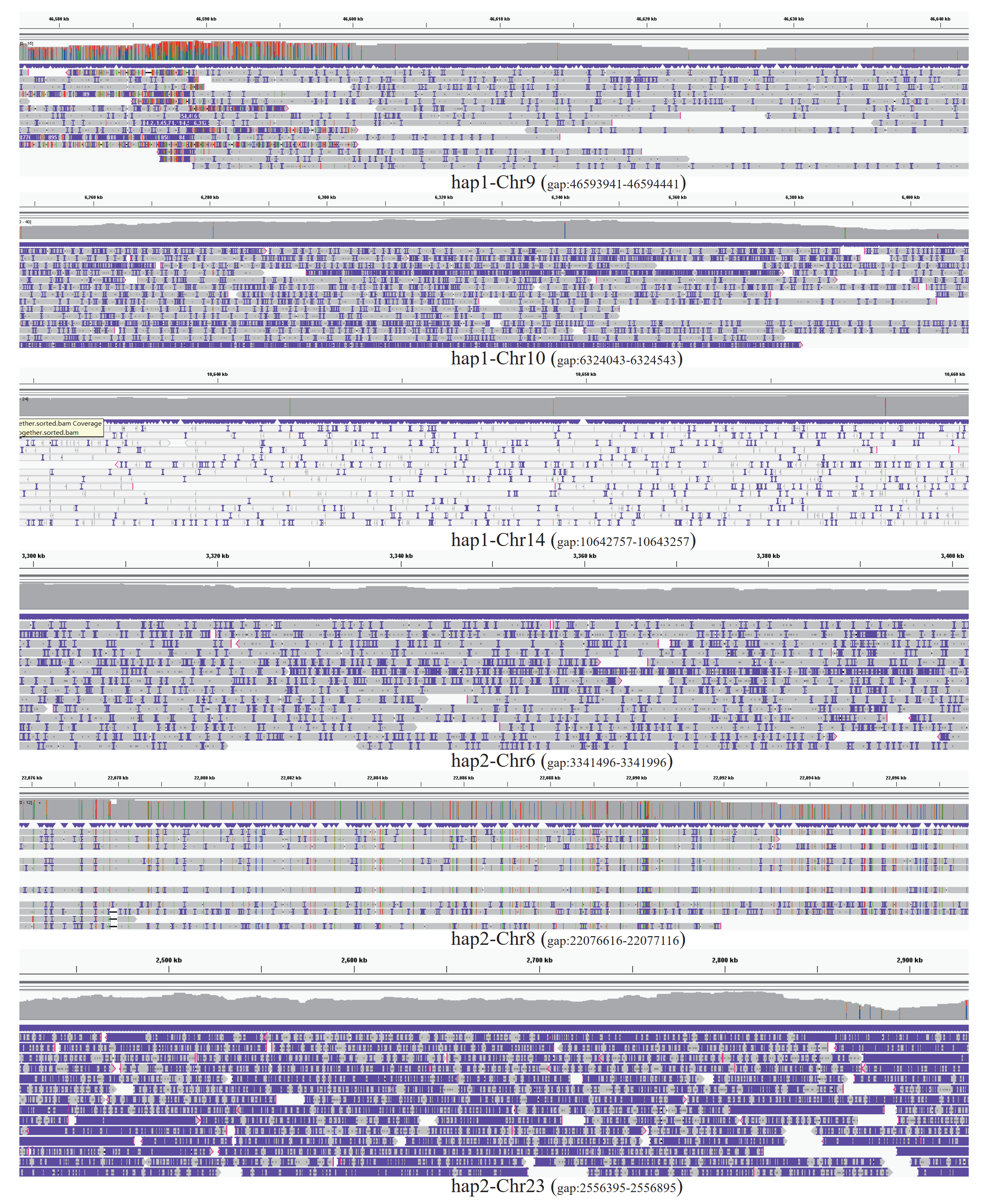


**Supplemental** Figure S4 Ultralong ONT reads coverage of six gaps in the two haplotypes.

Detailed information of gaps was within the brackets.


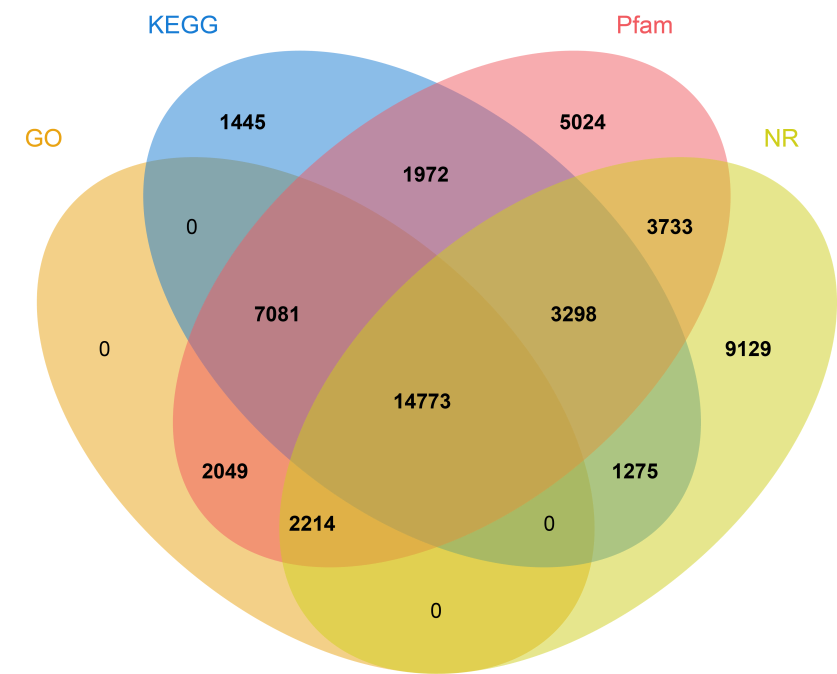


**Supplemental** Figure S5 The number of annotated genes in the *C. retusus* genome in databases


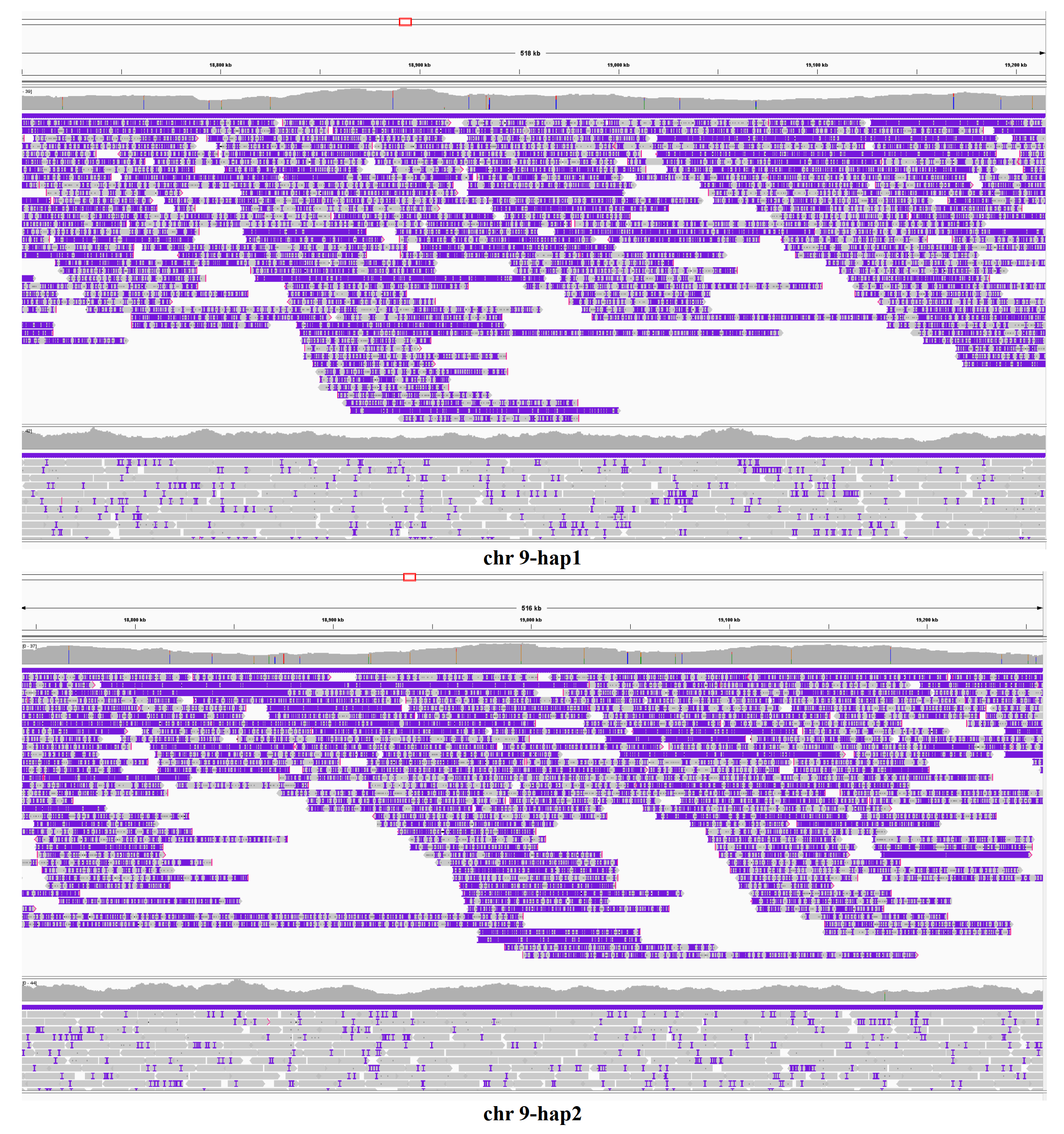


**Supplemental** Figure S6 The distribution of ONT reads surrounding the variant region on chromosome 9


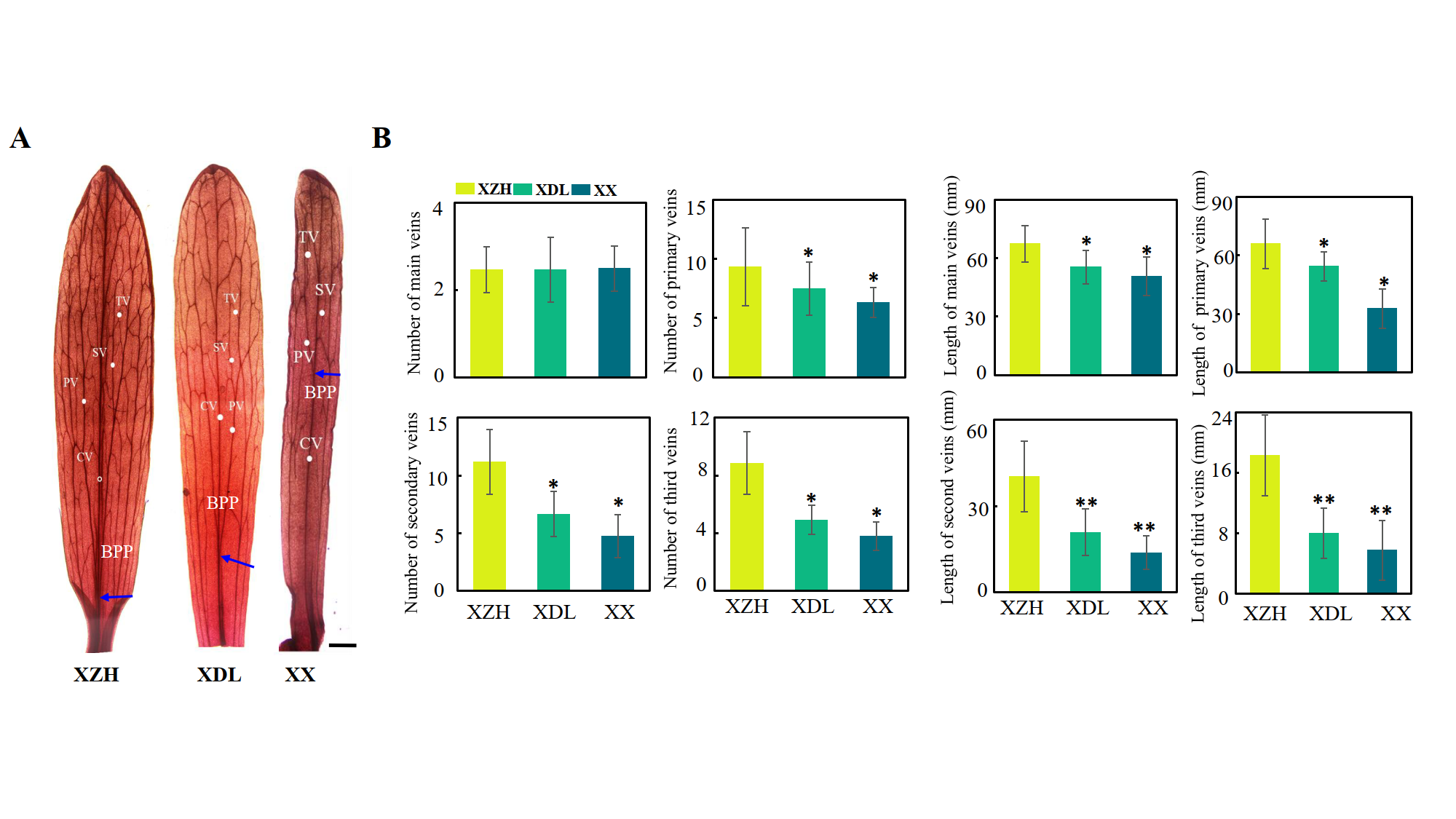


**Supplemental** Figure S7 Number and length of flower veins in three varieties of *Chionanthus retusus*.

(A) The vein transparency shows the difference flower veins of three varieties, TV, tertiary vein; PV, primary vein; SV, secondary vein; CV, chief vein; BPP, Bifurcation point position; Bar=5mm.

(B) Statistics of the number and leagth of flower veins in three varietie. Data are presented as means ± SD (n = 30). Asterisks indicate significant differences (t-test) compared to the XZH. ***p*< 0.01, **p*<0.05.


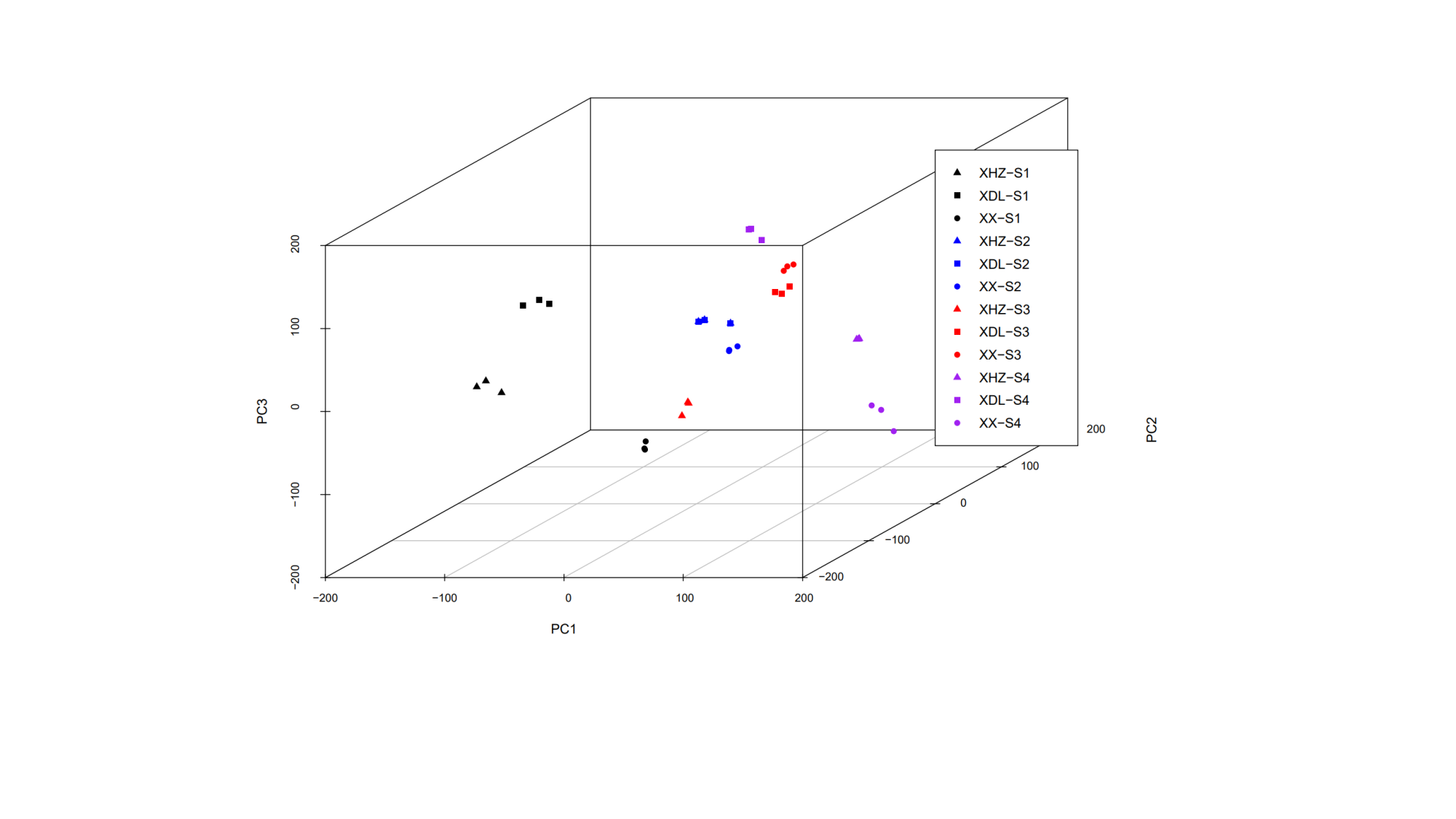


**Supplemental** Figure S8 Principal component analysis of correlation between different samples


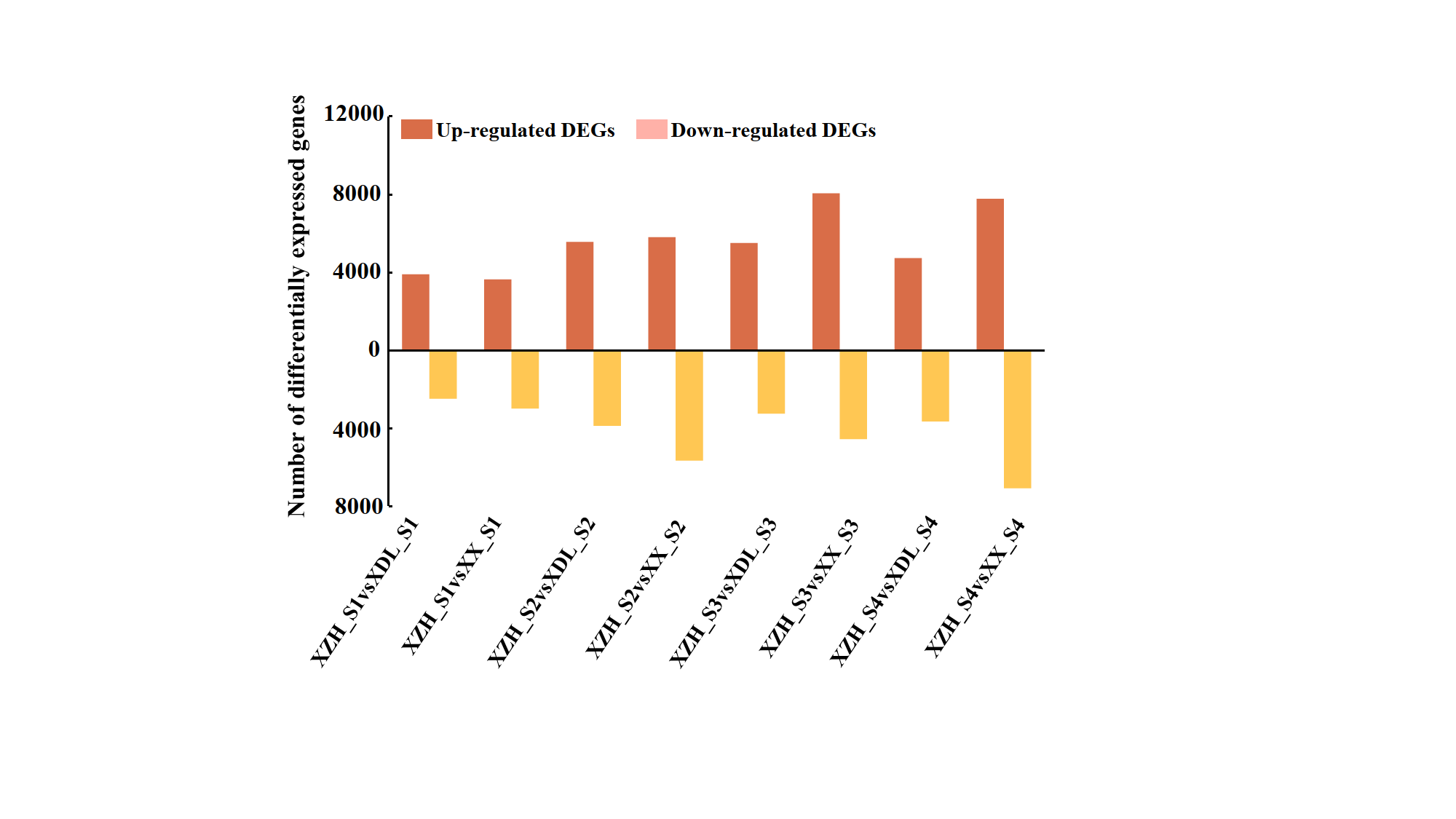


**Supplemental** Figure S9 The differentially expressed genes in different flower development stages of three varieties.


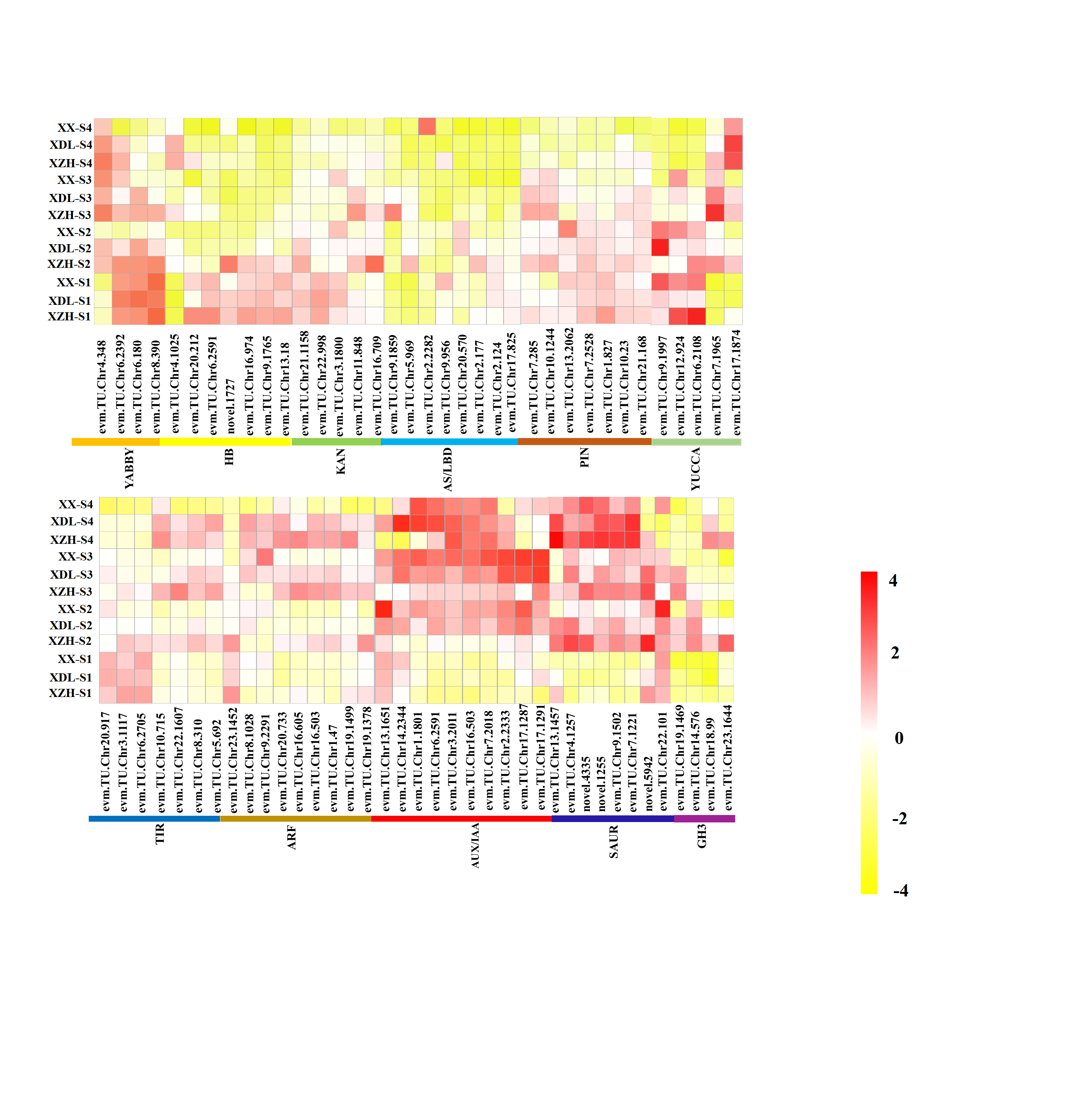


**Supplemental** Figure S10 Expression patterns of auxin and polarity related genes in flower at different development stages


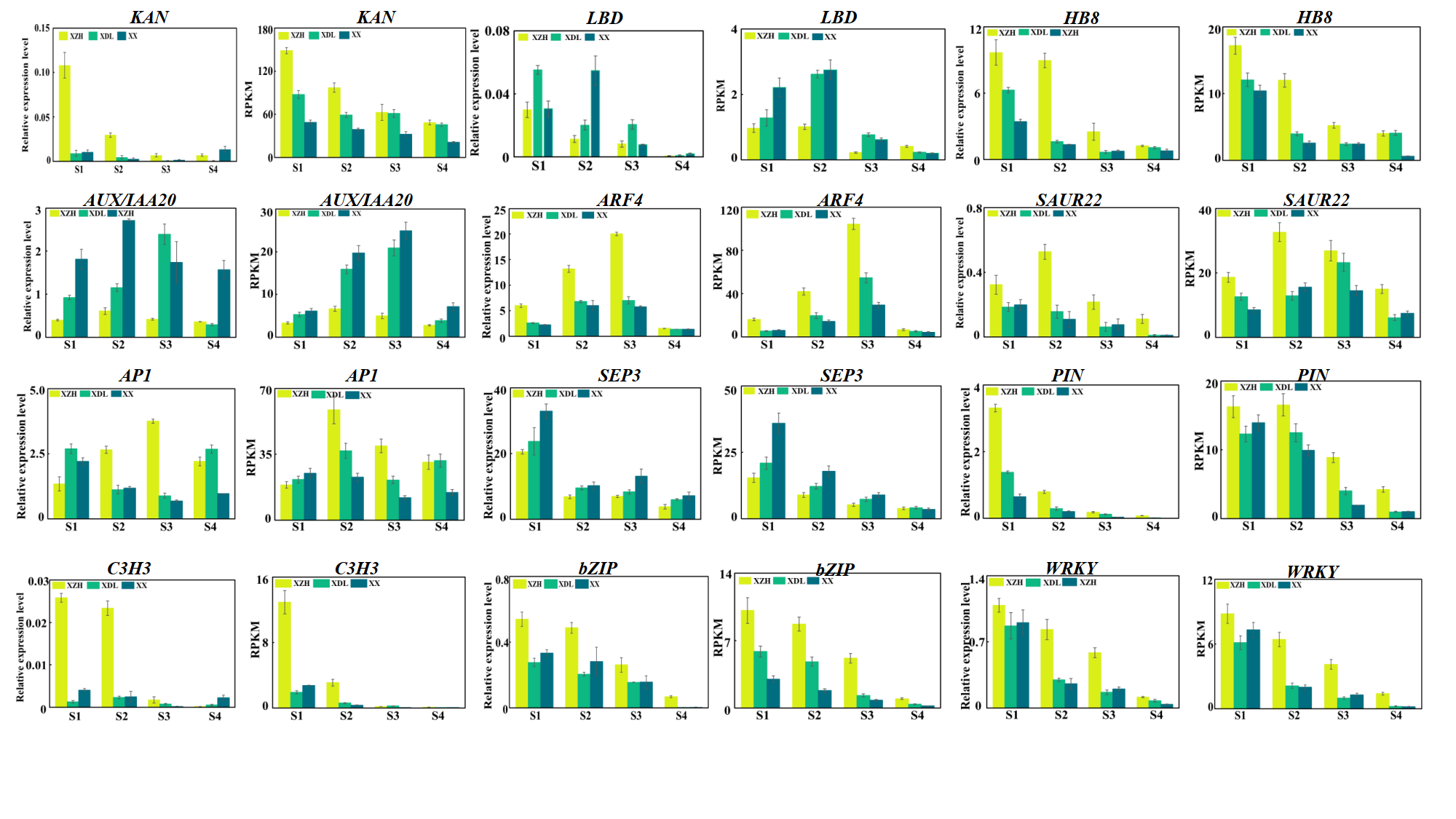


**Supplemental** Figure S11 Verification of expression patterns of genes related to petal vasculature.

UBC2 was used as an internal control. Data are presented as means ± SD (n = 3).


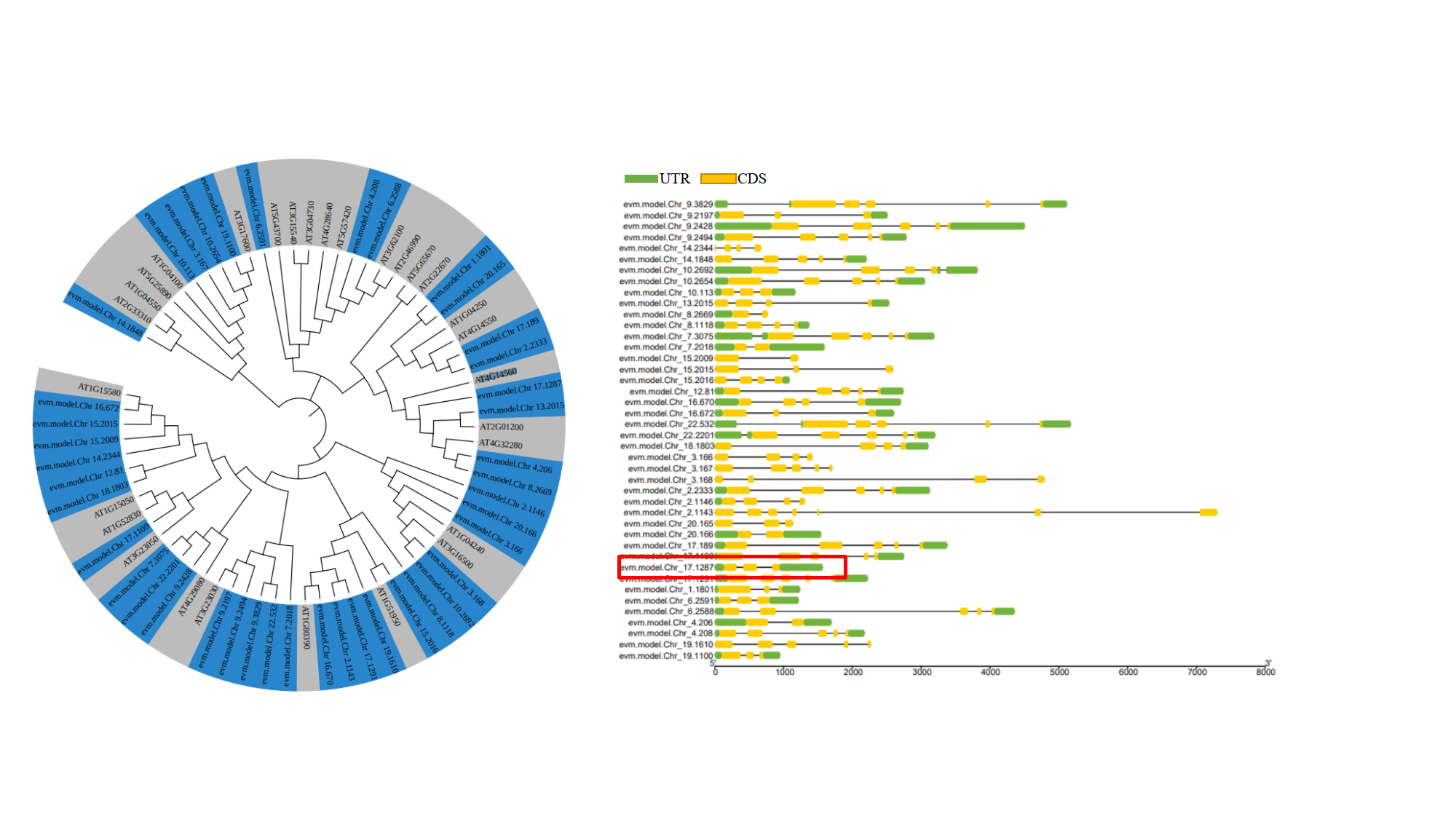


**Supplemental** Figure S12 Analysis of AUX/IAA gene family and gene structure in *Chionanthus retusus*

*
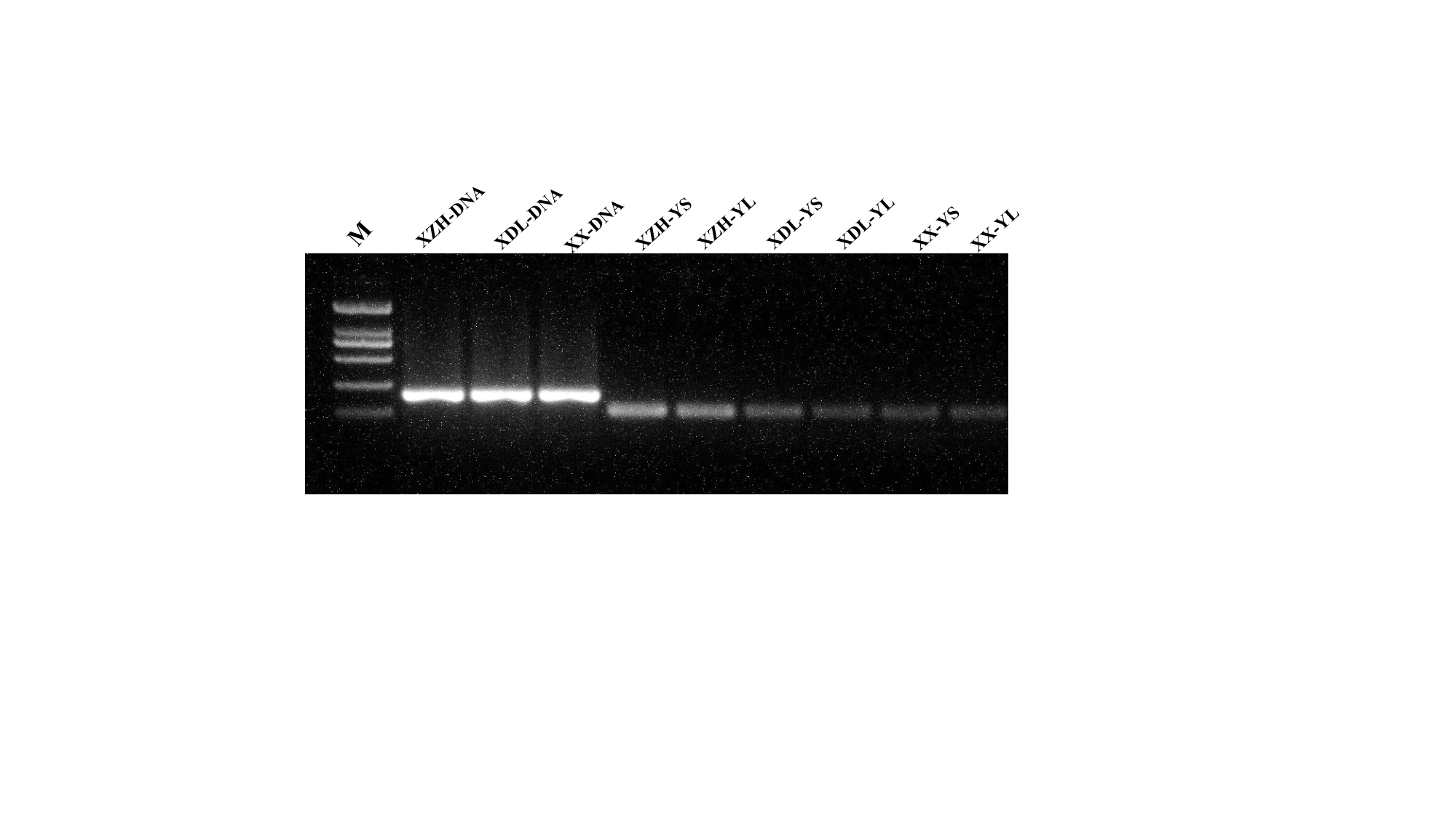
*

**Supplemental** Figure S13 *CrAUX/IAA20* only contains one transcript in other tissues of *C. retusus*.

YS, Young stem; YL, Young leaf; M: marker 2000.

*
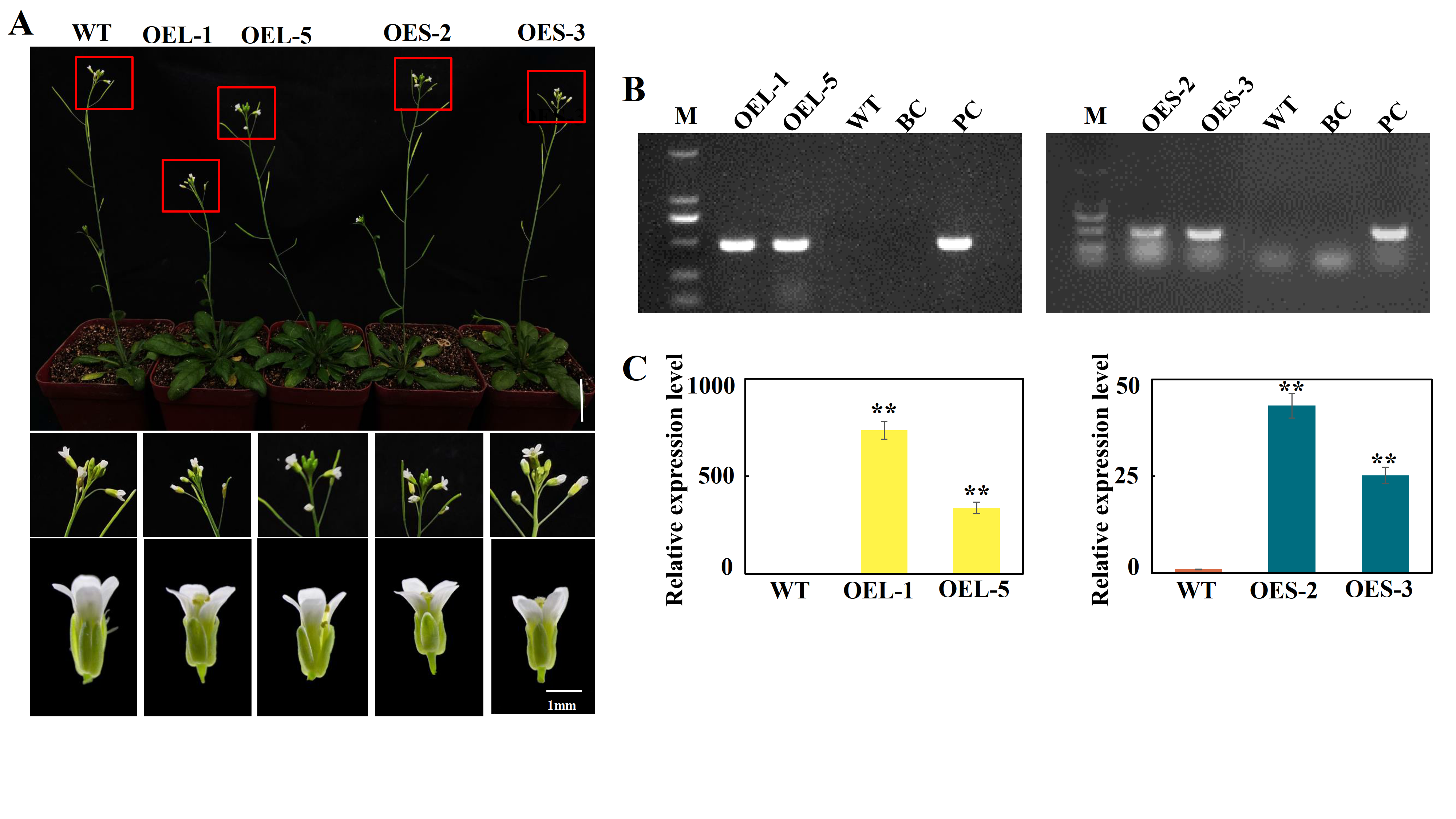
*

**Supplemental** Figure S14 Analysis of *CrAUX/IAA20-L* and *CrAUX/IAA20-S* transgenic plants.

(A) Photograph and flower morphology of 1‐month‐old non‐transgenic control (WT), *CrAUX/IAA20-L* (OEL) and *CrAUX/IAA20-S* (OES) overexpression transgenic plants. Bar=1mm.

(B) PCR detection of transgenic plants *CrAUX/IAA20-L* and *CrAUX/IAA20-S*; M, DL2000; WT, negative control; BC, blank control; NC, positive control.

(C) Expression of *CrAUX/IAA20-L* and *CrAUX/IAA20-S* plants determined via reverse transcription quantitative polymerase chain reaction (RT-qPCR). *Actin* was used as an internal control. The expression of *CrAUX/IAA20-S* and *CrAUX/IAA20-L* in the WT was set as 1. Data are presented as means ± SD (n = 3). Asterisks indicate significant differences (t-test) compared to the WT. ** *p* < 0.01.


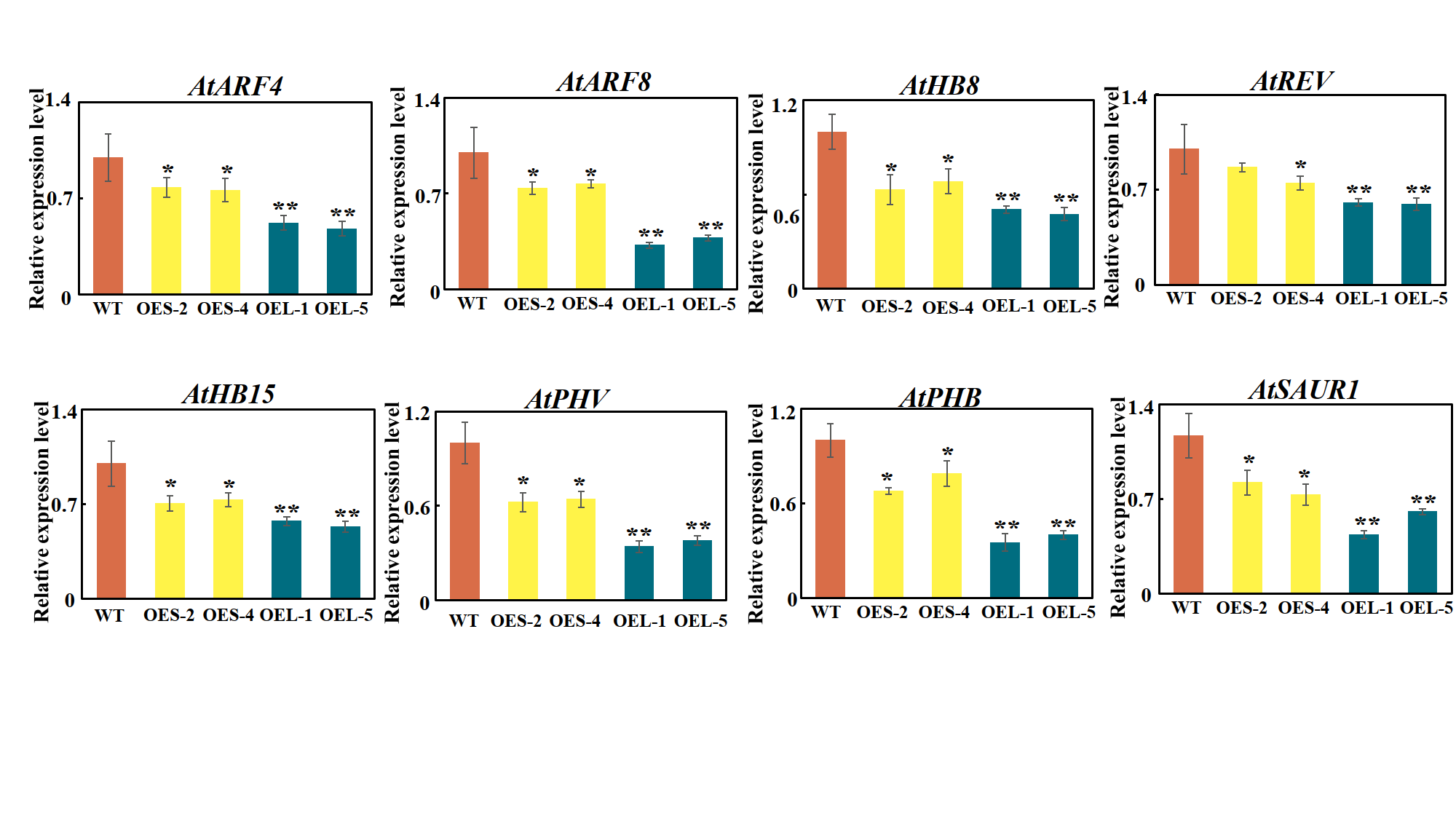


**Supplemental** Figure S15 Overexpression of *CrAUX/IAA20* can inhibit the expression of related genes in *Arabidopsis*.

*Actin* was used as an internal control. The expression of each gene in the WT was set as 1. Data are presented as means ± SD (n = 3). Asterisks indicate significant differences (t-test) compared to the WT. **p*<0.05, ***p*<0.01.

**Supplemental Tables**

Table S1. Summary of genome sequencing and read out for *C. retusus*

| Type | Insert size | Read length | Total raw data | Genome coverage |
| --- | --- | --- | --- | --- |
| PacBio reads | - | - | ~41.00G (pass data) | ~55.03X |
| Ultra-long ONT reads | - | - | ~71.00G (pass data) | ~52.07X |
| Illumina-HIC | 350bp | 150bp | ~76G | ~102.01X |
| Total | - | - | ~157.04G | ~157.04X |

Table S2. Survey statistic results of *C. retusus*.

| Sample | *k*-mer | *k*-mer number | *k*-mer depth | Genome size (Mb) | Heterozygous ratio (%) |
| --- | --- | --- | --- | --- | --- |
| *C. retusus* | 17 | 55,280,653,676 | 73 | 757 | 1.68% |

Table S3. Summary of Hi-C reads mapping to the *C. retusus*.

|  | Read number | Percentige |
| --- | --- | --- |
| Total Clean Pairs | 724,665,630 | 100.00% |
| Place Clean Pairs | 715,870,786 | 98.79% |
| Unplace Clean Pairs | 8,794,844 | 1.21% |

Table S4. Result of the evalutation of the *C. retusus* genome by BUSCO and LAI.

| Type | Number | Percent |
| --- | --- | --- |
| Complete BUSCOs (C) | 1602 | 99.20% |
| Complete and single-copy BUSCOs (S) | 1340 | 83.00% |
| Complete and duplicated BUSCOs (D) | 262 | 16.20% |
| Fragmented BUSCOs (F) | 7 | 0.40% |
| Missing BUSCOs (M) | 5 | 0.40% |
| Total BUSCO groups searched | 1614 | 100% |
| LAI | - | 16.2 |

Table S5. Gene numbers and features of *C. retusus*.

|  | *C. retusus* |
| --- | --- |
| Number of genes | 42,864 |
| Average gene length | 3241.17bp |
| Average cds length | 1320.46bp |
| Average exons per gene | 5.15 |
| Average exon length | 249.9bp |
| Longest exon | 7922bp |

Table S6. Basic statistical results of gene structure of closespecies of *C. retusus*.

| Species | Number | Average transcript length(bp) | Average CDS length(bp) | Average exons per gene | Average exon length(bp) |
| --- | --- | --- | --- | --- | --- |
| *C. retusus* | 42,864 | 3241.17 | 1320.46 | 5.15 | 249.9 |
| *P. trichocarpa* | 31,517 | 3,316.72 | 1,391.65 | 5.55 | 250.69 |
| *O. europaea* | 53,517 | 3,841.30 | 1,127.32 | 5.15 | 218.95 |
| *F. excelsior* | 38,684 | 3,918.15 | 1,165.68 | 5.24 | 222.28 |

Table S7. The statistical results of gene function annotation of *C. retusus*.

|  | Gene number | Percent (%) |
| --- | --- | --- |
| Nr | 28,516 | 66.53 |
| KEGG | 29,844 | 69.62 |
| GO | 26,117 | 60.93 |
| Pfam | 40,145 | 93.65 |
| Annotated | 41,352 | 96.47 |
| Total | 42,864 | - |

Table S8. The statistical results of non-coding RNA of *C. retusus*.

| Type | | number | Average length(bp) | Total length(bp) | % of genome |
| --- | --- | --- | --- | --- | --- |
| miRNA | | 731 | 108.91 | 80,590 | 0.011121 |
| tRNA | | 716 | 74.89 | 58,341 | 0.008051 |
| rRNA | rRNA | 7,237 | 320.25 | 2,317,643 | 0.32 |
|  | 18S | 840 | 1,719.87 | 1,444,693 | 0.2 |
|  | 28S | 3,188 | 138.34 | 441,037 | 0.060861 |
|  | 5.8S | 796 | 164.38 | 130,843 | 0.018056 |
|  | 5S | 356 | 124.77 | 301,070 | 0.041546 |
| snRNA | nRNA | 1,343 | 120.07 | 161,251 | 0.022252 |
|  | CD-box | 1,138 | 117.5 | 133,710 | 0.018451 |
|  | HACA-box | 60 | 128.6 | 7,716 | 0.001065 |
|  | splicing | 133 | 134.86 | 17,937 | 0.002475 |
|  | scaRNA | 12 | 157.33 | 1,888 | 0.000261 |
|  | Unknown | 0 | 0 | 0 | 0 |

Table S9. Summary of repeat contents in *C. retusus* genome.

|  | Denovo+Repbase | | TE Proteins | Combined TEs | | |
| --- | --- | --- | --- | --- | --- | --- |
|  | Length(bp) | % in Genome | Length(bp) | % in Genome | Length(bp) | % in Genome |
| DNA | 25,062,762 | 3.46 | 470,071 | 6.00 | 25,142,838 | 3.47 |
| LINE | 3,002,035 | 0.41 | 622,601 | 9.00 | 3,443,595 | 0.48 |
| SINE | 1,926 | 0 | 0 | 0.00 | 1,926 | 0 |
| LTR | 289,082,232 | 39.89 | 76,875,304 | 1061.00 | 295,316,955 | 40.75 |
| Unknown | 56,461,621 | 7.79 | 0 | 0.00 | 56,461,621 | 7.79 |
| Total | 363,423,783 | 50.15 | 77,967,752 | 1076.00 | 366,016,966 | 50.51 |

Table S10. Statistics of cluster numbers, length, GC content, *k-*mer content and ncRNAs of Single Chromosomes.

| Sequeues ID | GC_ratio | Sequeues Length | *k-mer* content | ncRNA |
| --- | --- | --- | --- | --- |
| Chr_1 | 33.88% | 25855673 | 0.9249405 | 659 |
| Chr_2 | 34.19% | 29093743 | 0.897759967 | 951 |
| Chr_3 | 34.17% | 31636222 | 0.914647281 | 936 |
| Chr_4 | 34.02% | 23852647 | 0.941443417 | 627 |
| Chr_5 | 34.14% | 26937202 | 0.926307296 | 748 |
| Chr_6 | 34.49% | 26121077 | 0.864782 | 597 |
| Chr_7 | 34.14% | 33436984 | 0.949184559 | 774 |
| Chr_8 | 34.08% | 33643664 | 0.953502176 | 849 |
| Chr_9 | 34.15% | 50044845 | 0.938472706 | 1229 |
| Chr_10 | 34.21% | 31600478 | 0.955313563 | 1042 |
| Chr_11 | 33.98% | 40902150 | 0.956404902 | 911 |
| Chr_12 | 34.26% | 31566960 | 0.9285105 | 721 |
| Chr_13 | 34.16% | 32910947 | 0.949814697 | 816 |
| Chr_14 | 34.21% | 40258976 | 0.939295341 | 1153 |
| Chr_15 | 34.08% | 31129154 | 0.93942275 | 850 |
| Chr_16 | 34.10% | 30086938 | 0.931701548 | 823 |
| Chr_17 | 34.13% | 27848144 | 0.963169107 | 725 |
| Chr_18 | 34.28% | 28374394 | 0.938009172 | 795 |
| Chr_19 | 34.12% | 24010324 | 0.90284396 | 687 |
| Chr_20 | 34.06% | 29885872 | 0.932927133 | 736 |
| Chr_21 | 33.88% | 26178592 | 0.916339407 | 607 |
| Chr_22 | 34.18% | 32480194 | 0.945317182 | 771 |
| Chr_23 | 35.28% | 20916540 | 0.876152 | 769 |
| Sequeues ID | GC_ratio | Sequeues Length | *k-mer* content | ncRNA |

Table S11. Monomer sequences of centromeres on different chromosomes of *C. retusus*.

| Chromosomes | Monomer sequences of centromeres |
| --- | --- |
| Chr_1 | TAATATTTAATTATTTAATTCAATTGTTTATATAATATTTAATATCGTATAATATTTAAT |
| Chr_2 | AAATATTAACTAAATAATTATAATTAAATATTACATAACCAATTACATTTATTACATAAACAATTAAATTACATAATTAAGTAAACAATTAAACG |
| Chr_3 | AATTAAATAATTAAATATTAATTAAATATTATACGATATTAAATATTATATAAACAATTG |
| Chr_4 | TTAATATTAAATTATTTAATTAAATTGTTTATATAATATTTAATATCGTATAATATTTAA |
| Chr_5 | AATTAAATAATTAAATATTAATTAAATATTATACGATATTAAATATTATATAAACAATTG |
| Chr_6 | TTTATATAATATTTAATATCGTATAATATTTAATTAATATTTAACTATTTAATTAAATTG |
| Chr_7 | ATAATTAAATATTAATTAAATATTATACGATATTAAATATTATATAAACAATTGAATTAA |
| Chr_8 | ATAATTAAATATTAATTAAATATTATACGATATTAAATATTATATAAACAATTGAATTAA |
| Chr_9 | TTTATATAATATTTAATATCGTATAATATTTAATTAATATTTAACTATTTAATTAAATTG |
| Chr_10 | TTAATATTTAATTATTTAATTAAATTGTTTATATAATATTTAATATCGTATAATATTTAA |
| Chr_11 | TAATATTTAATTAATTAATTAAATTGTTTATATAATATTTAATATCGTATAATATGTAAT |
| Chr_12 | ATAATTAAATATTAATTACATATTATACGATATTAAATATTATATAAACAATTTAATTAA |
| Chr_13 | TTAATATTTAATTATTTAATTCAATTGTTTATATAATATTTAATATCGTATAATATTTAA |
| Chr_14 | TTAATATTTAATTATTTAATTCAATTGTTTATATAATATTTAATATCGTATAATATTTAA |
| Chr_15 | ATAATTAAATATTAATTAAATATTATACGATATTAAATATTATATAAACAATTTAATTAA |
| Chr_16 | CCAAATTGAAGTTTAGAAATAGTTGAAGGATGAAATTGCTTTTAAATAATAGTTGGATGA |
| Chr_17 | ATAATTAAATATTAATTAAATATTATACGATATTAAATATTATATAAACAATTGAATTAA |
| Chr_18 | ATAATTTGATCCTCCAACTATCTAAATCATATAATTTGATCCTCCAACTATCTAAATCAT |
| Chr_19 | ATAATTTGATCCTCCAACTATCTAAATCATATAATTTGATCCTCCAACTATCTAAAACAT |
| Chr_20 | AATTAAATAATTAAATATTAATTAAATATTATACGATATTAAATATTATATAAACAATTG |
| Chr_21 | AATTGCTTTTAAACAATAGTTGGATGACCCAATTGAAGTTTCCTAAAGGTTGAAGGACTA |
| Chr_22 | GTTGGATGACCAAATTCGAATTTACAAATAGTTGAAGGATGAAATTGAATTTAAATTATA |
| Chr_23 | ATAATTAAATATTAATTACATATTATACGATATTAAATATTATATAAACAATTTAATTAA |

Table S12. The statistics of LTRs in the hap1 of *C. retusus* genome


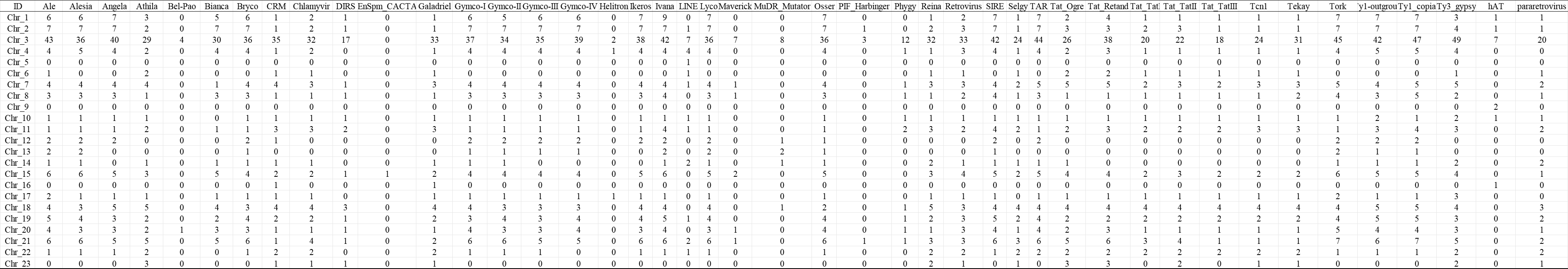


Table S13. Transcriptome data quality analysis.

| sample | library | raw_reads | clean_reads | error_rate | Q20 | Q30 | GC_pct |
| --- | --- | --- | --- | --- | --- | --- | --- |
| XZH_S1_1 | FRAS220182156-1r | 43296218 | 42318926 | 0.03 | 97.83 | 93.66 | 44.08 |
| XZH_S1_2 | FRAS220182156-2r | 45429514 | 44677938 | 0.03 | 97.96 | 93.96 | 43.86 |
| XZH_S1_3 | FRAS220182156-3r | 45698232 | 45139006 | 0.03 | 97.65 | 93.17 | 43.81 |
| XZH_S2_1 | FRAS220182157-1r | 46419544 | 45994362 | 0.02 | 98.11 | 94.3 | 43.47 |
| XZH_S2_2 | FRAS220182157-2r | 44550472 | 44257854 | 0.03 | 97.85 | 93.71 | 43.41 |
| XZH_S2_3 | FRAS220182157-3r | 45647584 | 45378794 | 0.03 | 98 | 94.04 | 43.3 |
| XZH_S3_1 | FRAS220182158-1r | 47507752 | 47217588 | 0.03 | 97.97 | 94 | 42.64 |
| XZH_S3_2 | FRAS220182158-2r | 44373844 | 44160968 | 0.03 | 97.94 | 93.9 | 42.79 |
| XZH_S3_3 | FRAS220182158-3r | 43975378 | 43094436 | 0.03 | 97.96 | 94.05 | 43.41 |
| XZH_S4_1 | FRAS220182159-1r | 47593552 | 46482194 | 0.03 | 98 | 94.13 | 43.17 |
| XZH_S4_2 | FRAS220182159-2r | 43926362 | 43050248 | 0.03 | 97.92 | 93.97 | 43.17 |
| XZH_S4_3 | FRAS220182159-3r | 45758070 | 44585076 | 0.03 | 97.84 | 93.78 | 43.05 |
| XDL_S1_1 | FRAS220182160-1r | 44305844 | 43353986 | 0.03 | 98 | 94.08 | 43.42 |
| XDL_S1_2 | FRAS220182160-2r | 46311370 | 45546932 | 0.02 | 98.14 | 94.4 | 43.56 |
| XDL_S1_3 | FRAS220182160-3r | 45160042 | 44681586 | 0.03 | 97.79 | 93.56 | 43.3 |
| XDL_S2_1 | FRAS220182161-2r | 44583812 | 44183952 | 0.03 | 97.96 | 93.98 | 43.65 |
| XDL_S2_2 | FRAS220182161-3r | 45998698 | 45521212 | 0.02 | 98.03 | 94.18 | 44.13 |
| XDL_S2_3 | FRAS220182161-4r | 46068854 | 45694180 | 0.03 | 98 | 94.15 | 44.27 |
| XDL_S3_1 | FRAS220182162-1r | 46654664 | 46362792 | 0.03 | 97.81 | 93.7 | 42.72 |
| XDL_S3_2 | FRAS220182162-2r | 46726478 | 46386168 | 0.03 | 97.73 | 93.57 | 42.32 |
| XDL_S3_3 | FRAS220182162-3r | 45929470 | 44771010 | 0.02 | 98.04 | 94.27 | 43.37 |
| XDL_S4_1 | FRAS220182163-1r | 45930154 | 44889960 | 0.03 | 97.81 | 93.68 | 43.1 |
| XDL_S4_2 | FRAS220182163-2r | 47102984 | 46062812 | 0.03 | 97.95 | 93.98 | 43.29 |
| XDL_S4_3 | FRAS220182163-3r | 46189084 | 45193404 | 0.02 | 98.09 | 94.35 | 42.26 |
| XX_S1_1 | FRAS220182164-1r | 47048598 | 46352512 | 0.02 | 98.12 | 94.29 | 43.46 |
| XX_S1_2 | FRAS220182164-3r | 47354198 | 46737722 | 0.03 | 97.96 | 93.95 | 43.13 |
| XX_S1_3 | FRAS220182164-2r | 45731666 | 44882686 | 0.03 | 97.91 | 93.82 | 43.41 |
| XX_S2_1 | FRAS220182165-1r | 45451128 | 44587662 | 0.02 | 98.11 | 94.35 | 44.53 |
| XX_S2_2 | FRAS220182165-2r | 46723570 | 45803052 | 0.02 | 98.08 | 94.24 | 43.48 |
| XX_S2_3 | FRAS220182165-3r | 49348690 | 48597506 | 0.03 | 98 | 94.03 | 43.76 |
| XX_S3_1 | FRAS220182166-1r | 44604194 | 44186208 | 0.03 | 97.82 | 93.76 | 42.62 |
| XX_S3_2 | FRAS220182166-2r | 44393884 | 43783142 | 0.02 | 98.06 | 94.3 | 42.71 |
| XX_S3_3 | FRAS220182166-3r | 44248618 | 42881272 | 0.03 | 97.78 | 93.72 | 43.43 |
| XX_S4_1 | FRAS220182167-1r | 46047150 | 44782720 | 0.03 | 97.92 | 93.99 | 43.79 |
| XX_S4_2 | FRAS220182167-2r | 46268836 | 45639796 | 0.03 | 97.75 | 93.63 | 43.78 |
| XX_S4_3 | FRAS220182167-3r | 45572466 | 43997198 | 0.03 | 97.81 | 93.72 | 44.01 |

| *YABBY*  Table S14. Expression patterns of genes related to vascular tissue development in petal. | | | | | | | | | | | | |
| --- | --- | --- | --- | --- | --- | --- | --- | --- | --- | --- | --- | --- |
| gene_id | XZH_S1 | XDL_S1 | XX_S1 | XZH_S2 | XDL_S2 | XX_S2 | XZH_S3 | XDL_S3 | XX_S3 | XZH_S4 | XDL_S4 | XX_S4 |
| evm.TU.Chr4.348 | 62.604 | 68.076 | 45.791 | 124.877 | 127.260 | 65.273 | 184.177 | 137.290 | 166.499 | 186.313 | 158.352 | 121.174 |
| evm.TU.Chr6.2392 | 141.678 | 160.433 | 139.603 | 144.896 | 95.979 | 55.681 | 116.359 | 86.521 | 109.513 | 122.645 | 106.459 | 38.083 |
| evm.TU.Chr8.2653 | 19.785 | 24.254 | 19.639 | 19.439 | 17.496 | 7.682 | 16.509 | 16.195 | 8.011 | 9.452 | 7.630 | 5.217 |
| evm.TU.Chr18.1938 | 135.789 | 85.385 | 127.137 | 62.899 | 8.577 | 2.559 | 26.933 | 2.699 | 1.395 | 0.416 | 4.452 | 0.671 |
| *AS/LBD* | | | | | | | | | | | | |
| gene_id | XZH_S1 | XDL_S1 | XX_S1 | XZH_S2 | XDL_S2 | XX_S2 | XZH_S3 | XDL_S3 | XX_S3 | XZH_S4 | XDL_S4 | XX_S4 |
| evm.TU.Chr2.2282 | 0.963 | 1.278 | 2.212 | 1.007 | 2.614 | 2.747 | 0.223 | 0.763 | 0.614 | 0.410 | 0.226 | 0.198 |
| evm.TU.Chr9.956 | 4.201 | 3.524 | 6.930 | 1.872 | 2.007 | 2.869 | 1.064 | 1.274 | 1.548 | 4.950 | 1.001 | 1.496 |
| evm.TU.Chr2.177 | 4.088 | 1.984 | 1.846 | 7.716 | 3.772 | 1.628 | 2.334 | 1.371 | 0.040 | 0.676 | 0.388 | 0.000 |
| evm.TU.Chr2.124 | 2.676 | 3.662 | 3.865 | 3.680 | 2.137 | 1.081 | 0.251 | 0.482 | 0.000 | 0.217 | 0.374 | 0.000 |
| evm.TU.Chr9.1859 | 0.867 | 0.819 | 0.209 | 1.338 | 0.811 | 0.328 | 12.799 | 3.449 | 0.951 | 1.390 | 1.671 | 0.159 |
| evm.TU.Chr5.969 | 0.700 | 0.349 | 0.122 | 10.513 | 4.943 | 3.013 | 4.346 | 3.822 | 1.896 | 0.404 | 0.235 | 0.689 |
| evm.TU.Chr17.825 | 12.284 | 12.446 | 9.084 | 7.631 | 7.510 | 5.633 | 4.048 | 1.715 | 0.072 | 0.684 | 0.902 | 0.000 |
| evm.TU.Chr20.570 | 1.170 | 2.120 | 2.052 | 1.691 | 4.460 | 4.305 | 1.375 | 0.836 | 0.565 | 0.326 | 0.656 | 0.000 |
| *HD-ZIP* | | | | | | | | | | | | |
| gene_id | XZH_S1 | XDL_S1 | XX_S1 | XZH_S2 | XDL_S2 | XX_S2 | XZH_S3 | XDL_S3 | XX_S3 | XZH_S4 | XDL_S4 | XX_S4 |
| evm.TU.Chr9.1765 | 23.561 | 18.559 | 14.474 | 13.839 | 6.109 | 3.092 | 1.240 | 0.932 | 0.955 | 0.416 | 0.213 | 0.000 |
| evm.TU.Chr4.1025 | 19.456 | 16.634 | 20.453 | 55.911 | 51.523 | 26.409 | 66.825 | 34.145 | 38.697 | 96.900 | 93.943 | 53.673 |
| novel.1727 | 22.923 | 21.886 | 13.627 | 41.171 | 8.381 | 7.424 | 6.200 | 4.427 | 4.721 | 9.902 | 6.094 | 13.191 |
| evm.TU.Chr6.2591 | 25.971 | 16.445 | 20.111 | 16.248 | 12.670 | 12.051 | 17.244 | 16.861 | 9.701 | 19.145 | 12.655 | 9.651 |
| evm.TU.Chr13.18 | 18.792 | 11.007 | 15.114 | 8.996 | 3.089 | 5.210 | 4.930 | 2.314 | 1.341 | 1.558 | 1.706 | 0.300 |
| evm.TU.Chr16.974 | 17.176 | 12.073 | 10.416 | 11.999 | 3.981 | 2.590 | 2.451 | 2.438 | 3.239 | 4.024 | 4.110 | 0.630 |
| evm.TU.Chr5.1462 | 31.191 | 20.591 | 22.022 | 8.314 | 7.280 | 5.364 | 10.784 | 6.552 | 7.171 | 9.064 | 6.011 | 2.560 |
| *KAN* | | | | | | | | | | | | |
| gene_id | XZH_S1 | XDL_S1 | XX_S1 | XZH_S2 | XDL_S2 | XX_S2 | XZH_S3 | XDL_S3 | XX_S3 | XZH_S4 | XDL_S4 | XX_S4 |
| evm.TU.Chr22.998 | 9.990 | 10.462 | 9.262 | 5.444 | 6.126 | 5.759 | 4.786 | 5.402 | 5.957 | 4.243 | 5.732 | 4.625 |
| evm.TU.Chr21.1158 | 7.464 | 9.136 | 6.883 | 11.234 | 7.712 | 5.062 | 3.425 | 3.442 | 3.511 | 2.218 | 2.994 | 1.343 |
| evm.TU.Chr11.848 | 8.523 | 11.759 | 10.608 | 6.414 | 7.419 | 11.359 | 4.903 | 5.394 | 10.232 | 5.013 | 5.932 | 2.415 |
| evm.TU.Chr21.1964 | 62.461 | 61.090 | 32.139 | 97.013 | 58.974 | 38.827 | 148.341 | 87.531 | 48.937 | 48.419 | 45.797 | 20.948 |
| evm.TU.Chr16.709 | 2.500 | 2.063 | 2.215 | 9.422 | 2.713 | 2.637 | 3.340 | 1.839 | 1.372 | 2.740 | 1.491 | 1.089 |
| *TAA1* | | | | | | | | | | | | |
| gene_id | XZH_S1 | XDL_S1 | XX_S1 | XZH_S2 | XDL_S2 | XX_S2 | XZH_S3 | XDL_S3 | XX_S3 | XZH_S4 | XDL_S4 | XX_S4 |
| evm.TU.Chr9.1767 | 4.248 | 1.425 | 2.746 | 9.650 | 6.365 | 6.890 | 59.322 | 23.855 | 18.193 | 13.163 | 47.261 | 26.362 |
| evm.TU.Chr5.1270 | 8.855 | 2.525 | 0.535 | 18.249 | 3.739 | 0.766 | 27.294 | 12.482 | 4.544 | 7.989 | 6.949 | 1.420 |
| evm.TU.Chr9.1066 | 2.795 | 3.208 | 2.488 | 5.908 | 9.322 | 14.810 | 3.679 | 9.776 | 5.293 | 8.734 | 10.304 | 9.968 |
| evm.TU.Chr5.1269 | 1.009 | 2.154 | 1.569 | 6.921 | 6.186 | 2.973 | 2.308 | 4.219 | 3.966 | 6.549 | 8.536 | 11.378 |
| *YUCCA* | | | | | | | | | | | | |
| gene_id | XZH_S1 | XDL_S1 | XX_S1 | XZH_S2 | XDL_S2 | XX_S2 | XZH_S3 | XDL_S3 | XX_S3 | XZH_S4 | XDL_S4 | XX_S4 |
| evm.TU.Chr7.1965 | 1.028 | 1.014 | 0.485 | 10.506 | 4.776 | 4.204 | 20.778 | 11.509 | 6.646 | 7.536 | 4.716 | 3.191 |
| evm.TU.Chr12.924 | 4.015 | 1.801 | 2.951 | 1.522 | 1.735 | 2.921 | 1.245 | 1.875 | 2.757 | 0.280 | 0.459 | 0.186 |
| evm.TU.Chr9.1997 | 0.596 | 0.692 | 1.360 | 0.393 | 1.760 | 1.150 | 0.332 | 0.342 | 0.029 | 0.073 | 0.035 | 0.030 |
| evm.TU.Chr17.1874 | 9.813 | 4.561 | 4.711 | 13.838 | 9.446 | 5.916 | 14.021 | 12.474 | 5.690 | 24.171 | 25.936 | 17.397 |
| evm.TU.Chr6.2108 | 7.807 | 1.939 | 4.469 | 4.069 | 2.124 | 2.748 | 1.528 | 1.322 | 0.475 | 0.217 | 0.328 | 0.000 |
| *PIN* | | | | | | | | | | | | |
| gene_id | XZH_S1 | XDL_S1 | XX_S1 | XZH_S2 | XDL_S2 | XX_S2 | XZH_S3 | XDL_S3 | XX_S3 | XZH_S4 | XDL_S4 | XX_S4 |
| evm.TU.Chr7.2528 | 16.264 | 12.295 | 13.932 | 16.519 | 12.475 | 9.909 | 8.881 | 4.017 | 1.941 | 4.242 | 0.983 | 0.994 |
| evm.TU.Chr7.2923 | 9.791 | 5.155 | 4.461 | 12.940 | 6.549 | 5.184 | 20.620 | 14.011 | 8.096 | 2.100 | 3.330 | 0.380 |
| evm.TU.Chr10.2546 | 17.081 | 15.139 | 12.468 | 17.935 | 11.328 | 10.854 | 15.031 | 11.299 | 4.777 | 10.666 | 8.340 | 0.614 |
| evm.TU.Chr1.827 | 9.348 | 5.056 | 5.978 | 3.960 | 3.783 | 3.806 | 1.684 | 1.898 | 1.254 | 1.552 | 0.555 | 0.747 |
| evm.TU.Chr21.168 | 58.908 | 50.401 | 38.132 | 50.365 | 48.748 | 55.366 | 52.789 | 54.904 | 35.489 | 40.949 | 10.815 | 6.583 |
| evm.TU.Chr10.1244 | 13.936 | 10.754 | 3.434 | 30.849 | 13.725 | 11.881 | 35.084 | 20.839 | 20.254 | 7.112 | 2.680 | 3.860 |
| evm.TU.Chr13.2062 | 4.012 | 4.398 | 7.061 | 3.828 | 4.585 | 19.240 | 0.932 | 3.472 | 2.315 | 0.336 | 0.850 | 1.468 |
| *SAUR* | | | | | | | | | | | | |
| gene_id | XZH_S1 | XDL_S1 | XX_S1 | XZH_S2 | XDL_S2 | XX_S2 | XZH_S3 | XDL_S3 | XX_S3 | XZH_S4 | XDL_S4 | XX_S4 |
| novel.5942 | 18.634 | 12.633 | 8.561 | 32.672 | 12.904 | 15.619 | 26.900 | 23.237 | 14.522 | 15.037 | 6.151 | 7.482 |
| evm.TU.Chr9.1502 | 3.978 | 5.371 | 4.182 | 50.349 | 37.447 | 17.636 | 55.005 | 30.180 | 32.340 | 119.032 | 87.403 | 29.045 |
| novel.4335 | 10.292 | 6.539 | 9.455 | 44.975 | 17.921 | 17.301 | 41.338 | 17.108 | 16.270 | 53.166 | 30.320 | 46.396 |
| novel.1255 | 18.828 | 9.084 | 11.662 | 75.056 | 64.175 | 25.691 | 126.952 | 102.659 | 31.516 | 328.553 | 236.592 | 170.204 |
| evm.TU.Chr4.1257 | 0.923 | 1.068 | 1.327 | 8.099 | 6.011 | 2.672 | 3.673 | 5.752 | 3.912 | 6.415 | 4.411 | 5.345 |
| evm.TU.Chr7.1221 | 3.156 | 4.807 | 2.714 | 12.623 | 8.294 | 6.922 | 14.068 | 8.550 | 10.230 | 26.680 | 27.488 | 14.569 |
| evm.TU.Chr22.101 | 9.295 | 10.029 | 11.828 | 10.899 | 13.379 | 31.328 | 5.281 | 9.330 | 7.665 | 1.512 | 0.923 | 12.565 |
| evm.TU.Chr13.1457 | 5.129 | 2.141 | 0.439 | 13.682 | 10.370 | 1.102 | 4.019 | 1.121 | 1.345 | 47.913 | 23.306 | 5.729 |
| *GH3* | | | | | | | | | | | | |
| gene_id | XZH_S1 | XDL_S1 | XX_S1 | XZH_S2 | XDL_S2 | XX_S2 | XZH_S3 | XDL_S3 | XX_S3 | XZH_S4 | XDL_S4 | XX_S4 |
| evm.TU.Chr18.99 | 4.090 | 0.981 | 1.226 | 29.819 | 18.563 | 5.153 | 14.384 | 8.892 | 7.422 | 62.579 | 30.936 | 18.040 |
| evm.TU.Chr23.1644 | 3.764 | 5.199 | 4.727 | 18.265 | 6.780 | 1.771 | 5.497 | 4.041 | 1.502 | 12.819 | 3.261 | 3.372 |
| evm.TU.Chr19.1469 | 5.141 | 4.992 | 2.467 | 15.686 | 15.342 | 5.231 | 25.519 | 20.676 | 6.833 | 7.051 | 6.627 | 3.038 |
| evm.TU.Chr14.576 | 7.752 | 3.682 | 2.384 | 60.573 | 55.298 | 36.716 | 21.755 | 11.216 | 7.039 | 9.421 | 5.512 | 7.032 |
| *TIR1* | | | | | | | | | | | | |
| gene_id | XZH_S1 | XDL_S1 | XX_S1 | XZH_S2 | XDL_S2 | XX_S2 | XZH_S3 | XDL_S3 | XX_S3 | XZH_S4 | XDL_S4 | XX_S4 |
| evm.TU.Chr20.917 | 45.048 | 55.082 | 52.818 | 30.365 | 31.453 | 37.516 | 28.127 | 35.001 | 30.475 | 23.387 | 24.216 | 10.538 |
| evm.TU.Chr3.1117 | 76.179 | 61.660 | 51.561 | 56.742 | 31.541 | 25.088 | 42.024 | 28.848 | 26.891 | 24.127 | 23.762 | 10.800 |
| evm.TU.Chr6.2705 | 42.881 | 30.855 | 40.601 | 23.470 | 13.805 | 10.113 | 14.633 | 8.424 | 9.398 | 5.674 | 9.184 | 2.760 |
| evm.TU.Chr8.310 | 51.594 | 50.454 | 45.238 | 121.056 | 80.209 | 44.406 | 114.664 | 108.652 | 59.811 | 123.999 | 112.798 | 25.079 |
| evm.TU.Chr5.692 | 14.757 | 11.400 | 13.939 | 34.667 | 17.854 | 19.081 | 60.258 | 34.928 | 23.961 | 34.535 | 60.486 | 7.505 |
| evm.TU.Chr22.1607 | 21.724 | 20.305 | 21.507 | 30.590 | 19.057 | 18.232 | 60.773 | 27.559 | 19.663 | 33.684 | 29.064 | 7.921 |
| evm.TU.Chr10.715 | 19.113 | 14.605 | 16.523 | 31.039 | 17.433 | 12.198 | 45.372 | 19.484 | 14.096 | 63.467 | 48.259 | 28.667 |
| *ARF* | | | | | | | | | | | | |
| gene_id | XZH_S1 | XDL_S1 | XX_S1 | XZH_S2 | XDL_S2 | XX_S2 | XZH_S3 | XDL_S3 | XX_S3 | XZH_S4 | XDL_S4 | XX_S4 |
| evm.TU.Chr20.733 | 16.159 | 5.390 | 5.951 | 42.036 | 19.736 | 14.430 | 104.432 | 54.641 | 29.355 | 226.663 | 152.232 | 44.393 |
| evm.TU.Chr8.1028 | 0.421 | 0.986 | 1.159 | 0.679 | 1.418 | 1.230 | 0.657 | 2.016 | 1.590 | 2.330 | 2.710 | 0.000 |
| evm.TU.Chr16.605 | 18.510 | 10.037 | 9.225 | 19.739 | 10.765 | 7.955 | 52.780 | 25.399 | 12.185 | 56.031 | 18.533 | 13.298 |
| evm.TU.Chr16.503 | 13.316 | 10.003 | 12.486 | 28.259 | 12.338 | 9.711 | 53.695 | 27.724 | 15.630 | 47.847 | 40.673 | 6.761 |
| evm.TU.Chr9.2291 | 29.177 | 31.703 | 40.942 | 26.671 | 28.979 | 41.548 | 28.299 | 45.568 | 87.251 | 52.849 | 56.027 | 22.061 |
| evm.TU.Chr1.47 | 4.680 | 5.860 | 6.513 | 24.599 | 10.138 | 4.615 | 45.207 | 25.276 | 8.360 | 44.878 | 30.711 | 5.987 |
| evm.TU.Chr6.1211 | 98.143 | 75.118 | 69.344 | 95.065 | 77.766 | 81.053 | 127.826 | 92.610 | 83.242 | 192.424 | 106.163 | 29.723 |
| evm.TU.Chr22.837 | 1.775 | 1.277 | 1.379 | 3.325 | 1.002 | 0.467 | 2.317 | 1.524 | 1.306 | 1.584 | 1.703 | 0.048 |
| evm.TU.Chr23.1452 | 1.980 | 1.062 | 0.997 | 1.913 | 0.504 | 0.509 | 0.677 | 0.473 | 0.000 | 0.079 | 0.039 | 0.000 |
| *AUX/IAA* | | | | | | | | | | | | |
| gene_id | XZH_S1 | XDL_S1 | XX_S1 | XZH_S2 | XDL_S2 | XX_S2 | XZH_S3 | XDL_S3 | XX_S3 | XZH_S4 | XDL_S4 | XX_S4 |
| evm.TU.Chr13.1651 | 8.664 | 9.290 | 9.792 | 7.270 | 11.346 | 22.456 | 5.626 | 8.888 | 11.168 | 2.538 | 10.855 | 2.745 |
| evm.TU.Chr16.503 | 8.031 | 19.126 | 11.155 | 30.908 | 111.014 | 123.116 | 70.503 | 169.061 | 246.528 | 211.302 | 226.293 | 169.230 |
| evm.TU.Chr7.2018 | 24.430 | 23.096 | 23.570 | 38.638 | 84.161 | 120.095 | 85.461 | 136.578 | 272.848 | 205.588 | 148.107 | 187.617 |
| evm.TU.Chr14.2344 | 46.780 | 52.307 | 60.719 | 43.656 | 70.546 | 62.288 | 48.085 | 88.600 | 88.881 | 23.978 | 121.061 | 55.470 |
| evm.TU.Chr3.166 | 3.383 | 4.360 | 5.766 | 7.989 | 18.027 | 18.056 | 15.476 | 20.213 | 44.098 | 51.903 | 47.670 | 33.093 |
| evm.TU.Chr17.1287 | 3.318 | 5.320 | 6.119 | 6.673 | 15.899 | 19.740 | 5.007 | 20.909 | 24.914 | 2.784 | 3.884 | 7.145 |
| evm.TU.Chr17.1291 | 3.300 | 13.600 | 6.800 | 10.716 | 17.571 | 20.422 | 28.069 | 50.485 | 51.794 | 9.071 | 10.137 | 16.277 |
| evm.TU.Chr1.1801 | 0.656 | 1.350 | 0.988 | 1.001 | 2.446 | 5.828 | 2.859 | 5.898 | 10.812 | 1.125 | 14.255 | 12.161 |
| evm.TU.Chr6.2591 | 13.848 | 16.698 | 21.438 | 49.233 | 128.535 | 113.024 | 76.020 | 154.785 | 210.720 | 80.841 | 343.497 | 237.083 |
| evm.TU.Chr2.2333 | 21.474 | 18.552 | 27.819 | 32.067 | 56.874 | 60.819 | 44.904 | 80.865 | 87.639 | 49.410 | 46.105 | 19.180 |

Table S15 Expression patterns of hub genes related to flower shape.

| GeneID | XZH_S1 | XDL_S1 | XX_S1 | XZH_S2 | XDL_S2 | XX_S2 | XZH_S3 | XDL_S3 | XX_S3 | XZH_S4 | XDL_S4 | XX_S4 |
| --- | --- | --- | --- | --- | --- | --- | --- | --- | --- | --- | --- | --- |
| evm.TU.Chr22.208 | 67.079 | 50.614 | 41.626 | 47.792 | 26.729 | 32.707 | 56.677 | 48.124 | 37.647 | 43.949 | 42.707 | 12.036 |
| evm.TU.Chr18.2321 | 21.068 | 20.240 | 19.972 | 36.946 | 60.256 | 69.488 | 51.695 | 83.624 | 149.307 | 140.285 | 152.447 | 161.494 |
| evm.TU.Chr14.64 | 16.159 | 5.390 | 5.951 | 42.036 | 19.736 | 14.430 | 104.432 | 54.641 | 29.355 | 226.663 | 152.232 | 44.393 |
| evm.TU.Chr17.1287 | 3.318 | 5.320 | 6.119 | 6.673 | 15.899 | 19.740 | 5.007 | 20.909 | 24.914 | 2.784 | 3.884 | 7.145 |
| evm.TU.Chr22.1266 | 10.292 | 6.539 | 9.455 | 44.975 | 17.921 | 17.301 | 41.338 | 17.108 | 16.270 | 53.166 | 30.320 | 46.396 |
| evm.TU.Chr22.515 | 33.627 | 22.923 | 21.886 | 41.171 | 8.381 | 7.424 | 8.200 | 4.427 | 4.721 | 13.191 | 6.094 | 9.902 |
| evm.TU.Chr18.1961 | 0.700 | 0.349 | 0.122 | 10.513 | 4.943 | 3.013 | 4.346 | 3.822 | 1.896 | 0.404 | 0.235 | 0.689 |
| evm.TU.Chr10.604 | 2.500 | 2.063 | 2.215 | 9.422 | 2.713 | 2.637 | 3.340 | 1.839 | 1.372 | 2.740 | 1.491 | 1.089 |

Table S16. The primers were used in the study.

| Primer name | Sequence (5'-3') | Application |
| --- | --- | --- |
| ARF-F | ATGAAGGCCACCACTGCCGGAG | Cloning |
| ARF-R | CTAATTTACACCATTATCAGAGCTGCTACAAGCT | Cloning |
| AUX/IAA-F | ATGGCTCACGAATATCATCTGAATCTTAAG | Cloning |
| AUX/IAA-R | TTACTCCCAAGGAACATCTCCAACAAG | Cloning |
| SAUR-F | ATGTCTGCATGCAACAAAATCCACCAC | Cloning |
| SAUR-R | TTACCAATTAGAAACTCCATGTAGAAGCGGC | Cloning |
| HB-F | ATGGGTGTTGATGATTTATGCAATACAAGCC | Cloning |
| HB-R | TTAACAAGCTGCTGATTGATTGTTGAAAGGA | Cloning |
| LBD-F: | ATGAGTGGTGGTCAGAATGGGAGTGG | Cloning |
| LBD-R | TCACTGCGAAGTGGAAGGCCTGAAT | Cloning |
| KAN-F | ATGGAGGGAAGTGGAGATAGTACAGAATATTCTAAG | Cloning |
| KAN-R | TCACAGAGTCAGATCCAGAGTACTCGCC | Cloning |
| ARFPro-F: | CCCCTACAAAAGTAATCCACACAAATA | Cloning |
| ARFPro-R: | GATGGGGTTCACCGGTTGAGCT | Cloning |
| AUX/IAAPro-F: | GGAGGGGCAAAGAGGCAAAAGA | Cloning |
| AUX/IAAPro-R: | CAAGTTTTCGGCAGATATTGTCAAGG | Cloning |
| SAURPro-F: | CTTTAGATAACAATGTGGATIGATTTTACT | Cloning |
| SAURPro-R: | GAACCCGTACTCCTCCTCGGCCCGGACCAG | Cloning |
| HBPro-F: | GGAGATTAAAATCAAATTTATCGTAAATTT | Cloning |
| HBPro-R: | GTCTATACAAATCGGTAGACGGATC | Cloning |
| LBDPro-F: | CCTTTGAAACTGAGGAAAATGCAAAAAGCC | Cloning |
| LBDPro-R: | AGCCTCATAACAAAGAGTAACCACGGCGTC | Cloning |
| KANPro-F: | GACTATTCTATTTAATATTTTAACTGATAG | Cloning |
| KANPro-R: | GAAGCGCAGATGAAGATCAGACGTCCACCG | Cloning |
| ProAUX/IAA20-F | gaccatgattacgccaagcttAAAAGAGGTCCCAATTATACATCTTG | Vector construction |
| ProAUX/IAA20-R | ggactgaccacccggggatccAATCACTTTTGATAGTTTCCTAAATGAAT | Vector construction |
| ProAUX/IAA20::GUS-F | gaccatgattacgccaagcttTTGGAGGGGCAAAGAGGC | Vector construction |
| ProAUX/IAA20::GUS-R | ggactgaccacccggggatccAATCACTTTTGATAGTTTCCTAAATGAAT | Vector construction |
| AUX/IAA20-1OE-F | acgggggactctagaggatccATGGCTCACGAATATCATCTGAATC | Vector construction |
| AUX/IAA20-1OE-R | cgatcggggaaattcgagctcTTACTCCCAAGGAACATCTCCAA | Vector construction |
| PBI121-F | ACGGGGGACTCTAGAGGATCC | Vector construction |
| PBI121-R | CGATCGGGGAAATTCGAGCTC | Vector construction |
| CrAUX/IAA20-1-F | GGCTCTGCTGTAGTGAGTGGGT | Identify |
| CrAUX/IAA20-1-R | CGAAAAGGAGGCTCGTTGTCAG | Identify |
| CrAUX/IAA20-2-F | TTGCCCAAATACTTGCCGTG | Identify |
| CrAUX/IAA20-2-R | AACAGTATGTCGAGCTATTTTTTGACTTA | Identify |
| ARF4-RT-F | GCCACAGCGAACAGGTTGC | Realtime |
| ARF4-RT-R | AAACCGCCGTGAGTGCTTG | Realtime |
| AUX/IAA20-RT-F | AAGGCTCTAATAGTTGGATGGC | Realtime |
| AUX/IAA20-RT-R | GACCCTTTGTAGCCTTCCCT | Realtime |
| SAUR-RT-F | TTACGGCGGTGGAGGAAG | Realtime |
| SAUR-RT-R | GACTCATCGCAAGGAATAGCC | Realtime |
| HB-RT-F | AAGGTGGATTCTAACAACAGGG | Realtime |
| HB-RT-R | CAGGGCAGACTGGGCTTTA | Realtime |
| LBD-RT-F | AATGCTTCCAAAATGCTGCTC | Realtime |
| LBD-RT-R | GCCATTTCTGCTGATGTTTCC | Realtime |
| KAN-RT-F | CAGGGTATTTACAGAAGGCACA | Realtime |
| KAN-RT-R | CATCCATTTCGCAAGTCCA | Realtime |
| PIN-RT-F | GGAGCGGTGGTTGATGGG | Realtime |
| PIN-RT-R | GGTGAAGGCTCTGATGATGACT | Realtime |
| WRKY-RT-F | ACCTGAAGAAGTTTACGAGCCG | Realtime |
| WRKY-RT-R | TCAAGGTGGCTCCGCTCA | Realtime |
| bZIP-RT-F | AAGTTCCAATAACGAAACACGA | Realtime |
| bZIP-RT-R | TTCATTTGGATTACGGCTTTG | Realtime |
| SEP-RT-F | AACTTTCTGTTCTTTGCGATGC | Realtime |
| SEP-RT-R | ACCCAGTTCCAGTGCCTCA | Realtime |
| AP1-RT-F | GAGGACTTGGATTCCCAGAGC | Realtime |
| AP1-RT-R | CAGTTCAAAGGCTGGGGTAGA | Realtime |
| C3H-RT-F | CAGTAGCAACAATGGAATGGG | Realtime |
| C3H-RT-R | GCATCAGCACGAATCCATCA | Realtime |
| AUX/IAA20-L-RT-F | GGCTCTGCTGTAGTGAGTGGGT | Realtime |
| AUX/IAA20-L-RT-R | CGAAAAGGAGGCTCGTTGTCAG | Realtime |
| AtARF4-RT-F | TTGTTGGTGCGATGGGATG | Realtime |
| AtARF4-RT-R | GGGTCTAACCGACTCCTCAAA | Realtime |
| AtARF8-RT-F | AGCAATACCAAGCAATGTTAGC | Realtime |
| AtARF8-RT-R | GCTGCGGAAGATTCTCACTC | Realtime |
| AtHB8-RT-F | TGACCCCTCAACATCAGCC | Realtime |
| AtHB8-RT-R | GGCTTCATCCCAGGCATTT | Realtime |
| AtHB15-RT-F | CTTGATGGAGGAGAATGACAGG | Realtime |
| AtHB15-RT-R | CGGAACCACGAAGGACGAT | Realtime |
| AtPHV-RT-F | TTCCAGAATCGCAGATGTCG | Realtime |
| AtPHV-RT-R | CCAGGCTTCATCCCAATCA | Realtime |
| AtPHB-RT-F | CTGGGTTCAGATGATTGGGATG | Realtime |
| AtPHB-RT-R | CGGATGAGAAACCCGCTTG | Realtime |
| AtREV-RT-F | GTCACAACTCCTCAGCATTCG | Realtime |
| AtREV-RT-R | GTATCTCAGGGTCCAGAAATCG | Realtime |
| AtSAUR1-RT-F | CGAGAATCGCAAGCCTCTT | Realtime |
| AtSAUR1-RT-R | ACAAACCTTGGAACAATGGG | Realtime |
| AtSAUR15-RT-F | TTTGAGGAGTTTCTTGGGTGC | Realtime |
| AtSAUR15-RT-R | GGTATTGTTAAGCCGCCCAT | Realtime |
| Actin-RT-F | ATCTCTATGGAAACATCGTTCTCAG | Realtime |
| Actin-RT-R | ATCCTCCGATCCAGACACTGTA | Realtime |
| CrUBC2-RT-F | GTGGAGTGTGGAGGATAAGGGTG | Realtime |
| CrUBC2-RT-R | TGTTGACAAAACCGAGGAAGGA | Realtime |
